# From conventional to biosensor-based detection of *Cryptosporidium* spp. and *Toxoplasma gondii* in food and water: Implications for food and water safety

**DOI:** 10.1016/j.fawpar.2026.e00341

**Published:** 2026-06-02

**Authors:** Munwar Ali, Xiaohui Liang, Ali Raza, Chang Xu, Tingting Sun, Qing He, Zizye Zhu, Kun Li, Lizeng Peng

**Affiliations:** a College of Veterinary Medicine, Nanjing Agricultural University, Nanjing 210095, China; b School of Life Sciences, Qilu Normal University, Jinan 250200, China; c China Energy Research Institute, Qilu University of Technology (Shandong Academy of Sciences), Jinan 250014, China; d School of Environmental and Rural Science, University of New England, Armidale, NSW, 2351, Australia; e Key Laboratory of Novel Food Resources Processing & Institute of Agro-Food Sciences and Technology, Shandong Academy of Agricultural Sciences, Jinan 250100, China

**Keywords:** Biosensors, Food, Water, Parasites, Detection, Hazards

## Abstract

Zoonotic food- and waterborne protozoan parasites (FWPPs) pose a significant global public health risk, causing substantial morbidity via contaminated fresh produce, water, and meat. Despite their notable impact, surveillance and detection technologies remain inadequate for high-priority protozoans such as *Cryptosporidium* spp. and *Toxoplasma gondii*, as current World Health Organization (WHO) and Food and Agriculture Organization (FAO) guidelines primarily focus on bacterial pathogens. This review evaluates the global burden of *Cryptosporidium* spp. and *T. gondii*, and highlights the limitations of conventional detection methods, justifying the forward-looking perspective on biosensors' applications in detecting protozoan parasites (PPs), and future strategies in this regard. The complex nature and varied transmission routes of these parasites, along with challenges such as culturing, sample preparation, and morphological similarities, complicate their detection by conventional methods like microscopy, serology, and molecular assays. Additionally, these limitations include time-intensive protocols, infrastructure requirements, cost, and lack of portability, which restrict their suitability for rapid, on-site detection. Recent advances in biosensor technology may offer rapid, sensitive, and accurate on-site detection of FWPPs, driving a paradigm shift toward a smart food safety system. This review highlights the potential of emerging biosensor technologies, especially electrochemical, optical, and piezoelectric (gravimetric) biosensors, for the detection of *Cryptosporidium* spp. and *T. gondii* in food and water. Integrating biosensors with nanotechnology, artificial intelligence, point-of-care systems and microfluidics to create portable, cost-effective biosensors may revolutionize food safety surveillance, mitigating the impact of FWPPs, and aligning with Hazard Analysis and Critical Control Points (HACCP) priorities to safeguard public health.

## Highlights

• Biosensors may enable rapid, on-site detection of protozoa in complex matrices.
• Electrochemical, optical, and piezoelectric biosensors can accurately detect a single oocyst.
• CRISPR/Cas12a integration with biosensor enhances specificity for parasite DNA detection.
• Biosensor adoption aligns with HACCP principles, revolutionizing food safety surveillance.
• Laboratory findings need to be translated to realistic large-volume/mass field samples.

## 1 Introduction

Foodborne diseases impose a substantial global health burden, affecting an estimated 600 million people and causing approximately 420,000 deaths annually (WHO, U.N, 2015; WHO, 2021). Parasitic infections represent a major component of this morbidity, with global estimates for 2010 showing around 400 million individuals infected; notably, foodborne transmission accounts for approximately 91.1 million cases (22%) and 52,000 deaths each year (Chalmers et al., 2020; Torgerson et al., 2015).

Among food- and waterborne protozoan parasites (FWPPs), *Cryptosporidium* spp. is a leading cause of diarrheal mortality, particularly in children under five (Khalil et al., 2018), while *T. gondii* causes significant morbidity through congenital infections, ocular disease, and life-threatening encephalitis in immunocompromised individuals (Montoya and Liesenfeld, 2004; Torgerson et al., 2015); both contaminate food and water with environmentally resistant oocysts (Fayer et al., 2000; Dubey, 2004). Furthermore, global climate shifts, including increased rainfall, elevate the risk of these PPs in water sources through terrestrial runoff and sewage bypasses, subsequently contaminating fruits and vegetables (Li et al., 2020a). Foodborne protozoan parasites pose a significant public health risk and are recognized in the International Organization for Standardization (ISO) standard (16140–2:2016/Amd 1:2024) (FAO, 2021; ISO (International Organization for Standardization), 2016a). Although the detection of *Cryptosporidium* spp. in food items is guided by ISO 18744:2016 (efficiency: 10–60% depending on matrix, limit of detection (LOD): ∼10–100 oocysts   L^−1^), it suffers considerable constraints for routine analytical applications, including the analysis of complex matrices such as fruit juice (Chalmers et al., 2020; ISO (International Organization for Standardization), 2016b). Additionally, no standard ISO method currently exists for the detection of *T. gondii* in food products (Marín-García et al., 2022), creating a critical regulatory void in parasite surveillance.

Unlike bacteria, *Cryptosporidium* spp. and *T. gondii* are difficult to cultivate in vitro*,* and their identification is challenging due to their complex life cycles, diverse hosts, and transmission routes, further complicated by overlapping characteristics shared across a vast range of protozoan species (Gabriël et al., 2022; Vinayak, 2020,Vinayak et al., 2020). Additionally, the diversity of food matrices in which parasites may occur, such as untreated milk, raw meat, contaminated vegetables, and fruits (ISO (International Organization for Standardization), 2016a, ISO (International Organization for Standardization), 2016b; FAO, 2021; WHO, U.N, 2015), together with variations in target identification, requirements for large sample volumes, and the complexities of sampling methods and associated preparation protocols, hinder accurate detection. Conventional detection methods for foodborne parasites such as *T. gondii* are constrained by challenges including variable DNA concentration and difficulties in parasite purification (Chalmers et al., 2020), often requiring days or weeks, involving significant time and financial investment (Di Nardo and Anfossi, 2020). Also, the detection of FWPPs is hindered by the inability to mass culture these protozoa, which limits the availability of standardized antigens. Moreover, antigenic variation across different life stages further restricts the development of reliable immunoassays (Feyziazar et al., 2022). To overcome these limitations, biosensor-based detection offers a promising alternative, utilizing diverse recognition elements, including aptamers (Iqbal et al., 2015; Hassan et al., 2021a; Siavash Moakhar et al., 2023), DNA probes (Gökce et al., 2016; Alves et al., 2017), synthetic receptors (Zhang et al., 2021; Sarkhosh et al., 2021), etc., bypassing the need for purified antigens while enabling rapid, sensitive, and portable analysis suitable for point-of-care (POC) testing (Luka et al., 2021; Yu et al., 2021; Lei et al., 2022). According to the International Union of Pure and Applied Chemistry (IUPAC), “a biosensor utilizes biochemical reactions, primarily involving enzymes, immune systems or cells, to detect a readable signal via electrical, thermal, magnetic or optical means”, underpinning POC testing (IUPAC, 2006).

This review briefly examines the public health threats posed by *Cryptosporidium* spp. and *T. gondii*, acknowledging that while conventional detection methods (microscopy, PCR, immunoassays) remain analytically robust in terms of sensitivity and specificity and are essential for validating emerging detection technologies, they are constrained by time-intensive protocols, infrastructure requirements, and portability. We discuss how biosensor-based detection can complement these established methods by addressing gaps in portability and real-time analysis. We also assess the suitability of selected biosensors for rapid, accurate, and on-site detection of these protozoa, as well as their associated challenges and future perspectives in food safety and surveillance.

## 2 Methodology for data searching and selection criteria

**Fig. 1:**
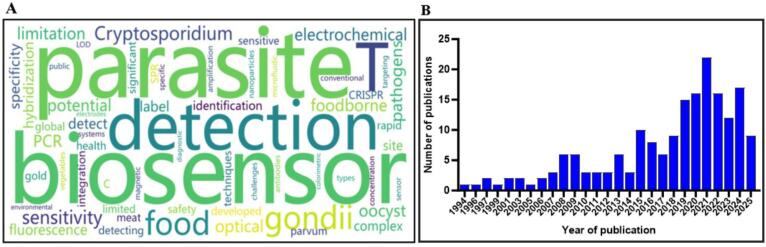
Schematic representation of (A) Word cloud visualization of key terms in the manuscript: It represents frequently used terms that encapsulate central themes of the given review article. The key terms are “biosensor, parasites detection, *Cryptosporidium*, *T. gondii*, food, sensitivity, and types of biosensors”, underscoring the basic focus of the whole manuscript. (B) Temporal distribution of reviewed publications: It represents the publication years of the included studies, with the majority between 2019 and 2025, reflecting a surge in research on the current topic in recent years. These trends highlight the growing scientific interest and emphasis on advancing the use of biosensor-based technologies for the detection of food- and waterborne protozoan parasites.

The data synthesized in this review were retrieved from electronic databases (Google Scholar, Science Direct, Web of Science, PubMed, SCOPUS) and targeted field-specific journals. Keywords included biosensor types like electrochemical, optical (including colorimetric, fluorescence, surface plasmon resonance (SPR)), piezoelectric (gravimetric), and thermal, *Cryptosporidium*, *T. gondii*, and FWPPs, public health, detection, diagnostic limitations, CRISPR/Cas12a integration with biosensors, advancements in biosensors, and future perspectives in biosensor development for FWPPs. Given the rapidly evolving nature of biosensor technology, emphasis was placed on publications from 2015 to 2025, while seminal earlier works establishing foundational methodologies (e.g., ISO validation standards, gold-standard bioassays) were included to provide necessary technical context. Reference selection prioritized studies reporting analytical validation data, including LOD, specificity, matrix testing, and regulatory guidelines (WHO/FAO/ISO); covering electrochemical, optical, and piezoelectric transduction platforms, while ensuring representation of both prototype development and validation in complex food/water matrices (Fig. 1A, B).

## 3 *T. gondii* and *Cryptosporidium* spp. as significant food- and waterborne hazards

As ∼70% of global water resources are linked to the agricultural sector, farm-based effluents frequently contaminate the natural ecosystems (Mateo-Sagasta et al., 2017; Wato, 2020). In Europe, 38% of water bodies are under significant pressure from agricultural pollution (Evans et al., 2019); hence, drinking water supplies and food products derived from food animals and fresh produce washed with contaminated water are at risk (WHO, U.N, 2015), elevating concerns about food- and waterborne pathogens, including PPs. An FAO/WHO Expert Committee reported 24 high-priority foodborne parasites globally (and 25 specifically for Europe in 2016), with *Cryptosporidium* spp. and *T. gondii* being of particular concern (FAO, 2021; WHO, U.N, 2015; Bouwknegt et al., 2018). These protozoa are often overlooked, yet they have caused a significant number of outbreaks in recent decades (Carstens et al., 2019). A recent systematic review and meta-analysis indicated that, based on available regional studies (predominantly from Asia and Africa), 41.22% of fruits and vegetables were contaminated with PPs, with Asia showing a notably high pooled prevalence of 57.12% (Eslahi et al., 2024), threatening global public health. *Cryptosporidium* spp. has been ranked fifth among 24 potential foodborne parasites (WHO, U.N, 2015). The Global Enteric Multicenter Study (GEMS) reported that it is the leading cause of diarrhea-associated mortality in children under five years of age in sub-Saharan Africa, second only to rotavirus (Chalmers et al., 2020; Kotloff et al., 2013). Sow et al. (2016) estimated approximately 202,000 *Cryptosporidium*-attributable deaths annually in children aged <24 months in sub-Saharan Africa and South Asia.

In terms of measurable healthcare burden, in the US, an estimated 822,000 cases occur annually due to cryptosporidiosis as the leading cause of zoonotic enteric illness (CDC (Centers for Disease Control and Prevention), 2019). Similarly, in India, *Cryptosporidium* spp. results in 3.9–7.1 million diarrheal episodes in children under five (Sarkar et al., 2014). In the Netherlands, the annual disease burden was estimated at 50,000 cases, 300 hospitalizations, and €19.2 million in total costs of illness (Monge et al., 2019). The Global Burden of Disease (GBD) study 2016 reported that globally, *Cryptosporidium* spp. infection was associated with 4,224,000 Disability Adjusted Life Years (DALYs) and 57,203 deaths in children under five (Troeger et al., 2018). Recent surveillance data demonstrate a significant increase in reported cases, with a marked rise in Spain (Martinez et al., 2024) and an enhanced detection revealing endemicity in Denmark (Larsen et al., 2025).

**Fig. 2:**
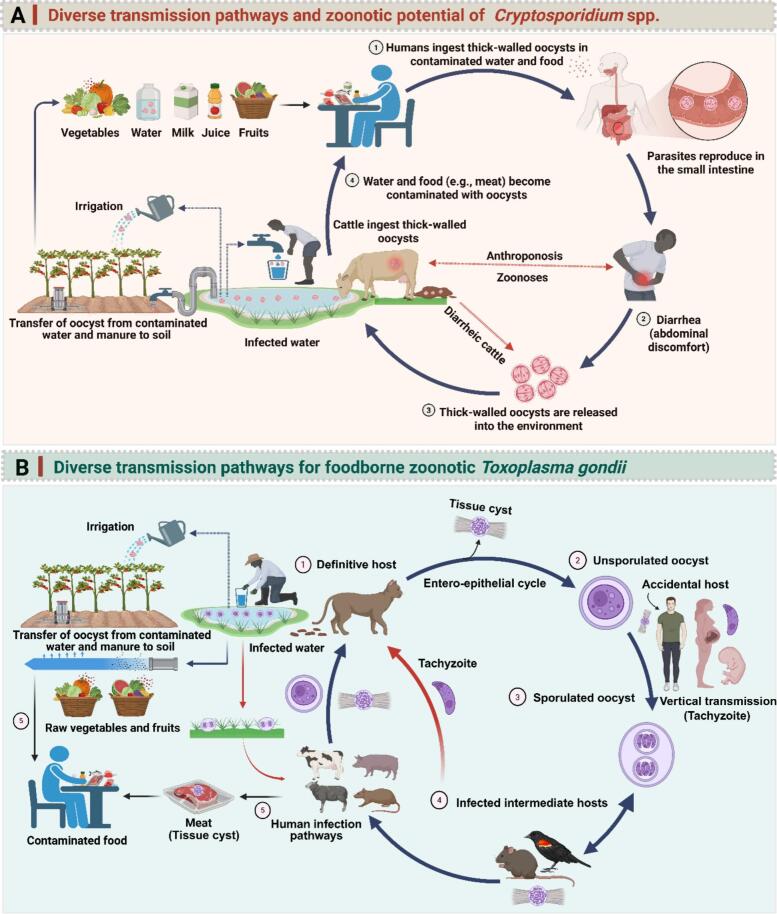
Transmission pathways of food- and waterborne protozoan parasites. (A) *Cryptosporidium* spp. transmission from infected hosts (livestock, humans) via oocyst shedding to water sources and agricultural produce (B) *T. gondii* transmission cycle: (1) Definitive hosts (felids) oocysts shed in feces; (2) Environmental sporulation and contamination of water/soil; (3) Ingestion by intermediate hosts (livestock, wildlife) or direct contamination of produce; (4) Human exposure via consumption of contaminated water, undercooked meat, and unwashed produce. Early detection at the environmental and food chain stages is critical for preventing human infection. Created in BioRender: https://BioRender.com/vowfth1, (License number: LU285QJ3XE).

Food and waterborne outbreaks have occurred worldwide due to protozoan parasites (Fig. 2A) (Augendre et al., 2023). In South America, a recent study found *Cryptosporidium* spp. in 6.6% of strawberries, blackberries, and peaches (González-Ramírez et al., 2024). In Southeast England, 17% of ready-to-eat vegetable samples tested positive for *C. parvum* DNA by nested PCR (Suleiman et al., 2024). In Spain, *Cryptosporidium* spp. was found in 6.8% of lettuce, spinach, cabbage, and strawberry samples and in 7.8% of lettuce samples in another study (Moreno-Mesonero et al., 2023; Trelis et al., 2022). In Australia, 10% of arugula samples were reported to be contaminated with *Cryptosporidium* and *Giardia* spp. (Ng-Hublin et al., 2018). In Kuwait, 5% of arugula samples collected in 2023–2024 were contaminated with parasites, including *Cryptosporidium* spp. (Al-Awadhi and Iqbal, 2025). In China, *Cryptosporidium* spp. and *Giardia* were detected in 1.7% of vegetable samples collected between 2015 and 2017 (Li et al., 2020b). In Morocco, *Cryptosporidium* spp. was found in 3.03% of fresh produce, including carrots, coriander, lettuce, parsley, and radish collected between 2017 and 2018 (Berrouch et al., 2020), emphasizing the need for enhanced surveillance through rapid and on-site detection. In Germany, a study of 23 cryptosporidiosis cases with travel history to Croatia in August and September 2023 reported that 93% could be attributed to swimming pools as the most likely source of infection (Schoeps et al., 2024). From 2009 to 2017, over 444 cryptosporidiosis outbreaks were reported in the US alone, with 15% originating from contact with infected cattle and 12.8% from contact with infected individuals in child care settings (Gharpure et al., 2019). The largest waterborne outbreak due to *Cryptosporidium* spp. occurred in Milwaukee, Wisconsin (USA) (Mac Kenzie et al., 1994), costing US$96.2 million, including medical expenditures of US $31.7 million and productivity losses of US$64.6 million (Corso et al., 2003). Globally, Karanis et al. (2007) identified 165 *Cryptosporidium*-associated waterborne outbreaks occurring up to 2004, Baldursson and Karanis (2011) reported 120 outbreaks (60.3% of total waterborne protozoan outbreaks) between 2004 and 2010, Efstratiou et al. (2017) documented 239 outbreaks between 2011 and 2016, and Bourli et al. (2023) identified 322 outbreaks between 2017 and 2022, indicating a significant health threat.

Similarly, *T. gondii* ranks among the highly prevalent zoonotic parasites of humans, infecting up to one-third of the global human population (Montoya and Liesenfeld, 2004; Hussain et al., 2017). Transmission occurs principally via two distinct routes: ingestion of environmentally resistant oocysts, shed by felid definitive hosts into the environment, including water sources and onto fresh produce, or consumption of viable tissue cysts in undercooked meat from infected intermediate hosts (Fig. 2B). Infectious doses can be as low as 1–10 oocysts (Augendre et al., 2023; Dubey, 2021). Waterborne outbreaks have been documented in municipal drinking water supplies (Bowie et al., 1997; Aramini et al., 1999) and in endemic settings (de Moura et al., 2006). Notably, the largest waterborne toxoplasmosis outbreak occurred in Victoria, British Columbia, Canada in 1995, with an estimated 2894–7718 individuals infected when the Humpback Reservoir serving 219,000 people became contaminated with *T. gondii* oocysts from infected cat and cougar feces washed into the water supply by heavy rainfall (Bowie et al., 1997; Aramini et al., 1999).

According to the FAO/WHO multicriteria global ranking of 14 foodborne parasites, *T. gondii* is the fourth most significant worldwide, based on disease burden measured in DALYs (FAO, 2021; WHO, U.N, 2015), and has been linked to several outbreaks. At the national level, it has ranked as the second most burdensome foodborne parasite in the United States (Batz et al., 2012; Hoffmann et al., 2012) and the leading one in the Netherlands (Havelaar et al., 2012). In a 2018 outbreak in the USA, 82% of individuals who consumed undercooked venison became ill (Schumacher et al., 2021). Similarly, in Quebec, Canada, an outbreak among deer hunters was linked to undercooked venison consumption, with 60% of the group developing symptoms (Gaulin et al., 2020). Ferreira et al. (2018) detected *T. gondii* in 12.9% of organic vegetable samples from Brazil, associating contamination with irrigation water sources and soil supplementation practices. In Europe, *T. gondii* was detected in 40% of watercress, lettuce, parsley, and different berry fruits in Portugal and Spain (Marques et al., 2020). *T. gondii* was also found in 3.6% of vegetable samples in China (Lass et al., 2019), and in 2020, 25% of sheep liver specimens were positive for *T. gondii* in Tunisia (Amdouni et al., 2024). All these studies clearly call for concern regarding early detection in food systems to control and prevent outbreaks.

## 4 Conventional detection methods and their associated limitations

### 4.1 Conventional detection methods for *Cryptosporidium* spp.

Presently, the detection of *Cryptosporidium* spp. in food items is guided by ISO 18744:2016 (ISO (International Organization for Standardization), 2016b), an international standard based on microscopy alone; however, this approach is not ideal for routine analysis due to limitations in species or genotype identification and oocyst infectivity assessment (Chalmers et al., 2020). Additionally, US-EPA 1623.1 for water is widely used as the gold standard for *Cryptosporidium* detection, but a critical drawback is its suboptimal recovery efficiency (≤ 50%), attributed to significant oocyst losses during centrifugation, post-filtration and elution, as documented in comparative studies using immunomagnetic separation followed by immunofluorescence microscopy (IMS-IFM) (Fradette et al., 2022). Furthermore, no validated method exists for the detection of *C. parvum* in frozen foodstuffs, creating a gap in food safety assessments (Åberg et al., 2015). The US-EPA 1623.1 method employs IMS-IFM for oocyst identification, typically achieving recovery efficiencies of ≤50% (< 10% to >80%); however, this approach is costly and requires skilled personnel for accurate interpretation (Efstratiou et al., 2017; Siwak et al., 2023). Hence, for on-site and real-time detection, more practical and cost-effective methods are needed.

Immunoassay-based methods for *Cryptosporidium* detection were developed to overcome the limitations associated with microscopy. The direct fluorescent antibody (DFA) assay, which uses fluorescein isothiocyanate-conjugated anti-*Cryptosporidium* monoclonal antibodies to bind the surface epitopes on oocysts and produce a yellow-green fluorescence, demonstrates 96–100% specificity and 98.5–100% sensitivity (Efstratiou et al., 2017). However, a drawback of DFA and other immunological assays is that commercially produced anti-*Cryptosporidium* monoclonal antibodies (mAbs) are raised against a limited pool of *C. parvum* isolates, and binding affinity may vary across species or genotypes, potentially making identification vague (Chalmers et al., 2020; Graczyk et al., 1996; Siwak et al., 2023). While DFA achieves 96–100% specificity, its detection limit typically ranges from 10^4^ to 10^5^ oocysts per gram (OPG) of environmental sample or fecal material, significantly higher than molecular methods (Efstratiou et al., 2017; Chalmers et al., 2020). Moreover, these standard methods cannot differentiate viable from non-viable oocysts without additional viability staining procedures (Brescia et al., 2009), exaggerating the actual public health risk. ELISA is predominantly used to detect *Cryptosporidium* spp. antigens in fecal samples, but due to the complexity of water samples and the typically low concentration of oocysts, ELISA is not directly applicable to water samples (Hassan et al., 2021b). Immunochromatographic lateral-flow assays have been developed for the rapid detection of *Cryptosporidium* in water and fecal samples; however, both ELISA and immunochromatographic strips may lead to false-positive results due to cross-reactivity with non-target organisms (Chalmers et al., 2020; Garcia and Shimizu, 1997; Siwak et al., 2023).

Also, molecular detection methods for *Cryptosporidium* spp. face multiple technical and methodological limitations. The primer design remains a critical challenge, particularly regarding real-time PCR, as non-specific amplifications from food matrices such as raspberries and iceberg lettuce have been shown to yield variable results in ring trials, necessitating post-PCR verifications such as sequencing to confirm target specificity, especially when targeting conserved regions like the 18S gene (Chalmers et al., 2020; Fradette et al., 2022). Furthermore, molecular methods, including nested PCR and RT-PCR, demand specialized equipment, technical expertise and costly reagents, limiting scalability (Mahmudunnabi et al., 2024; O'Leary et al., 2021). Analytically, PCR-based methods achieve detection limits of 1–10 oocysts per reaction in purified water samples; however, in complex food matrices (e.g., raspberries, lettuce), practical LODs often rise to 10^2^–10^3^ OPG due to extraction losses, PCR inhibition by humic acids and co-contaminants (Fradette et al., 2022; Mahmudunnabi et al., 2024).

### 4.2 Conventional detection methods for *T. gondii*

**Fig. 3:**
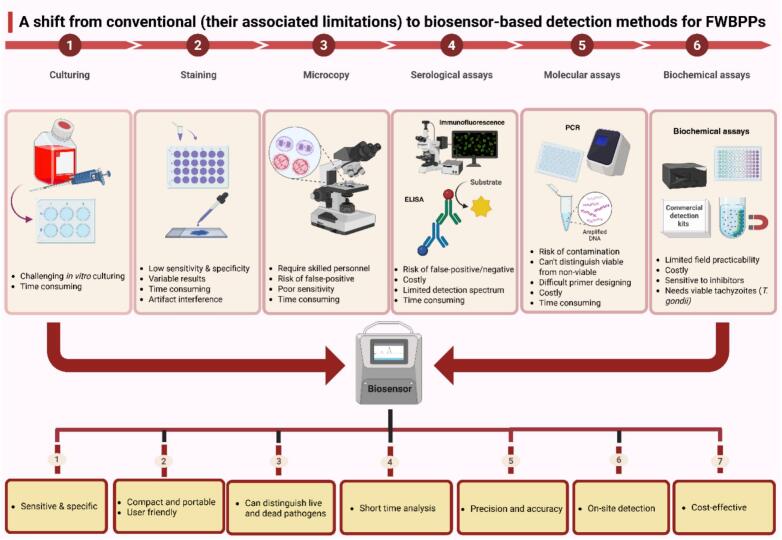
A detailed outline demonstrating the transition from conventional detection methods to a biosensor-based approach for FWPPs. The top panel highlights the conventional detection methods (from microscopy to biochemical assays) along with their associated limitations, while the bottom panel illustrates the benefits of biosensor-based technologies, emphasizing the significance of the above-mentioned shift in food safety and surveillance by overcoming the constraints of traditional detection techniques. Created in BioRender: https://BioRender.com/vf1vn1y, (License number: OS285QLYVL).

Despite the significant public health threat posed by *T. gondii,* standardized methods for detecting the parasite across diverse food matrices contaminated with different life stages (tachyzoites, bradyzoites, oocysts) remain unavailable (Marín-García et al., 2022). A gold standard test for confirming *T. gondii* viability in meat tissue is the bioassay, which recovers viable tachyzoites or bradyzoites. While the mouse or cat bioassay can theoretically detect as few as 10 tachyzoites, the 6-week incubation period and ethical constraints limit its practical utility for large-scale studies (Opsteegh et al., 2020). Cell culture methods (e.g., human foreskin fibroblasts) offer alternatives but exhibit lower sensitivity than bioassays (Opsteegh et al., 2020). In contrast, no equivalent method exists for oocysts, and their isolation and concentration from complex matrices such as raw vegetables remain hindered by the lack of precise and efficient laboratory techniques (Eslahi et al., 2024). Light microscopy enables identification but carries low sensitivity and requires skilled personnel (Liu et al., 2015) and risks false-positive results due to morphological similarities between oocysts and food-derived debris (Fig. 3) (Eslahi et al., 2024).

Molecular detection of *T. gondii* is limited by technical constraints. Magnetic capture qPCR (MC-qPCR), though validated for *T. gondii* tissue cysts in pork (Fig. 3) (Abhari et al., 2023; Algaba et al., 2017), remains underutilized. Conventional PCR targeting the B1 gene in water and meat indicated that 90% of samples were positive for *T. gondii* (Useche et al., 2022), with reported detection limits of 65.4 tachyzoites per 100 g of meat (Algaba et al., 2017; Useche et al., 2022). Assays targeting single-copy genes (e.g., B1) show reduced sensitivity compared to multi-copy targets (e.g., 529-bp repeat element) due to low genomic copy number, particularly in complex food matrices (Reischl et al., 2003). In addition, real-time PCR requires expensive instrumentation (Abhari et al., 2023). These PCR-based methods are primarily used for detection. In outbreak investigations, PCR detection was successfully applied during the 1995 Victoria, British Columbia waterborne toxoplasmosis outbreak to isolate and characterize *T. gondii* from contaminated reservoir water (Aramini et al., 1999; Bowie et al., 1997). Later, multilocus PCR-RFLP genotyping identified the type I strain responsible for the 2001 toxoplasmosis outbreak in Brazil linked to contaminated water (de Moura et al., 2006).

For detection in water, oocysts are typically concentrated by filtration, followed by molecular detection, though standardized methods are lacking compared to *Cryptosporidium* (Karanis et al., 2007; Dumètre et al., 2013). These constraints emphasize the need for biosensor-based approaches to identify PPs in contaminated water and food matrices.

## 5 A shift from conventional to biosensor-based detection for PPs

### 5.1 Overview, working principle, and classification of biosensors

**Fig. 4:**
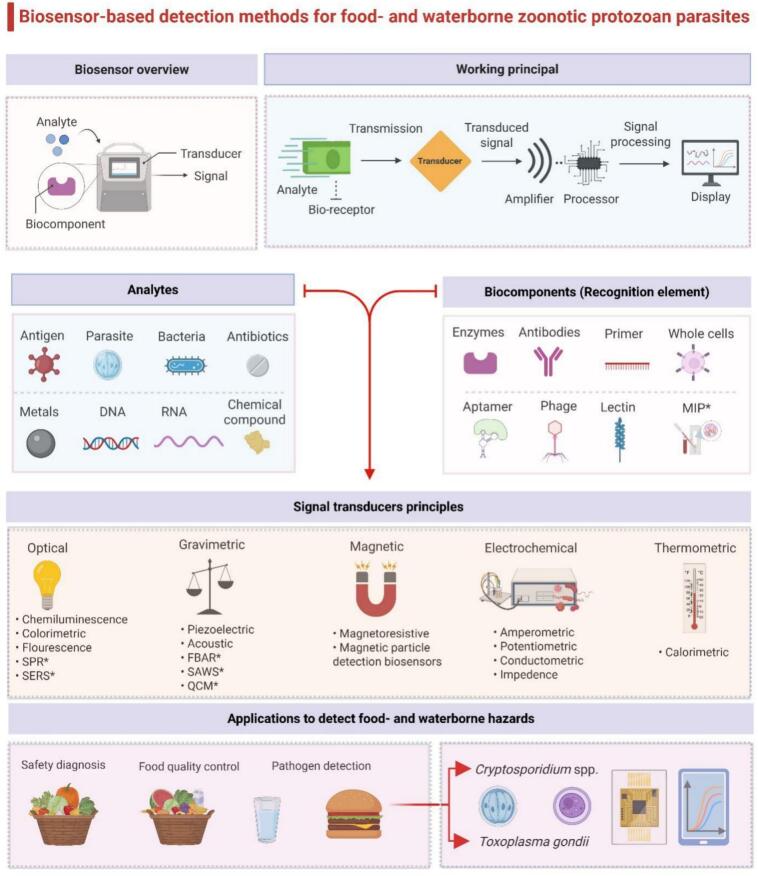
Schematic presentation of biosensor-based detection methods to detect FWPPs. Biosensor systems integrate recognition elements (enzymes, antibodies, aptamers, phages, etc.) with transducers (optical, electrochemical, gravimetric, magnetic) to convert analytes (antigens, DNA, parasites) into measurable signals. Applications span food safety diagnosis, pathogen detection, and water hazard monitoring. Created in BioRender: https://BioRender.com/sj3iim2, (HO285QML8O).

Biosensors are analytical devices that can overcome the limitations of conventional testing methods by enabling rapid, precise, and sensitive detection of pathogens. Portable and cost-effective biosensors provide real-time monitoring of food quality and manufacturing process, combining high sensitivity, rapidity, and accuracy for tracking pathogens or their metabolic byproducts (Turasan and Kokini, 2021; Yasmin et al., 2016). The recognition element (RE) identifies analytes in complex samples, the transducer translates the biological response into a measurable output, and the processor presents interpretable data (Fig. 4).

Biosensors are classified by RE, including antibiotic (Byrne et al., 2009; Sande et al., 2021), antibody (Byrne et al., 2009; Crivianu-Gaita and Thompson, 2016), aptamer (Crivianu-Gaita and Thompson, 2016; Hianik et al., 2024; Xiao et al., 2021), bacteriophage (Al-Hindi et al., 2022), DNA/RNA (Techakasikornpanich et al., 2024), enzyme (Vaidya and Annapure, 2019), lectin (Mi et al., 2021), molecularly imprinted polymer (MIP) (Zhang et al., 2021), and whole cell (Wu et al., 2021) types. They can also be classified by transducer type: electrochemical (amperometric, impedimetric, potentiometric, and voltammetric) (Singh et al., 2021), gravimetric (Cali et al., 2020), optical (e.g., SPR) (Chen and Wang, 2020), magnetic (Turasan and Kokini, 2021; Yasmin et al., 2016) or thermal (Ramanathan and Danielsson, 2001) (Fig. 4). Biosensor classifications also include genosensors (DNA/RNA detection), immunosensors (proteins/antibodies), and aptasensors (Davis and Higson, 2012; Felix and Angnes, 2018; Hosu et al., 2018), thereby encompassing a wide range of recognition strategies. Although advancements in biosensor development for detecting foodborne pathogens (bacteria, viruses) and toxins (mycotoxins) have been made in recent years (Banakar et al., 2022; Eyvazi et al., 2021; Hjort et al., 2024), a research gap persists regarding the use of biosensor technology for the detection of zoonotic PPs like *Cryptosporidium* spp. and *T. gondii* in food and water matrices.

### 5.2 Types of biosensors aimed at detecting *Cryptosporidium* spp. in food and water samples

#### 5.2.1 Electrochemical biosensors

Electrochemical sensors, a pivotal analytical tool, function by interfacing chemical solutions with sensor systems to generate electrical signals proportional to analyte concentrations. Due to the small size of *Cryptosporidium,* usually a chemical agent is used to amplify the electrochemical signal to a detectable level, as the organism covers a small portion of the electrode surface and imparts only a slight difference in current variations at the electrode-electrolyte surface. It involves a biorecognition element (e.g., aptamer, enzyme, antibody) that binds to target analyte (e.g., *Cryptosporidium* oocysts), followed by transduction, which includes different methods like electrochemical impedance spectroscopy (EIS), which measures changes in charge transfer resistance (Luka et al., 2022); voltammetry (square wave voltammetry/differential pulse voltammetry), which recognizes current changes resulting from redox reactions (Iqbal et al., 2015); and capacitance, which detects dielectric changes at the electrode-solution interface when the analyte binds to immobilized receptors (Luka et al., 2019). Finally, the electrical output (current and impedance) is quantified and correlated with analyte concentration with high sensitivity. Current platforms integrate nanomaterials, such as gold nanoparticles (AuNPs), to amplify signals (Iqbal et al., 2019)*.* These approaches are beneficial due to their ability to detect electrochemical responses at specified reduction potentials, ensuring specificity and precision (Hasanzadeh et al., 2018; Nemati et al., 2023; Safarpour et al., 2020; Singh et al., 2021).

##### 5.2.1.1 Impedance spectroscopy-based electrochemical biosensor

**Fig. 5:**
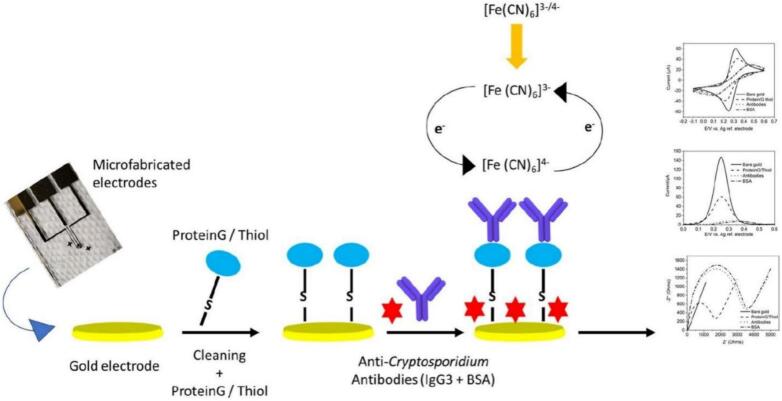
The modified sensor surface was analyzed by CV, SWV, and EIS. In the absence of *Cryptosporidium* spp. oocysts, the [Fe(CN)₆]^3−^/^4−^ redox probe diffuses through the monolayer to access the working electrode (WE) surface, where its oxidation/reduction generates a detectable charge transfer current. Conversely, the presence of oocysts blocks the monolayer, increasing the film's resistance and hindering electron transfer. This disruption is quantified via EIS, which reveals elevated impedance due to oocysts-induced interference. Adapted from (Luka et al., 2022), Sci Rep 12:6957. https://doi.org/10.1038/s41598-022-10765-0. Licensed under CC BY 4.0 (https://creativecommons.org/licenses/by/4.0/).

Due to their cost-effectiveness, miniaturized EIS systems are used to construct biosensor chips. A significant feature of these types of biosensors is label-free detection. In this biosensor type, impedance is measured by applying an AC potential to an electrochemical cell, and the current and resistance across the system are measured according to Ohm's law (Khare et al., 2022). In this context, Luka et al. (2022) developed a fast, cost-effective, on-chip electrochemical sensor with high sensitivity for detecting *Cryptosporidium* oocysts. The equipment includes a gold electrode functionalized with anti-*Cryptosporidium* mAbs (IgG3 subclass) immobilized via thiolated protein G (Fig. 5). By measuring changes in charge-transfer resistance, this biosensor achieves a detection limit of 20 oocysts in a 5 μL detection volume. While environmental waters require pre-concentration from larger volumes (standard practice in parasitological monitoring), this sensitivity enables rapid, label-free confirmation of *Cryptosporidium* spp. in concentrated water samples or high-risk matrices, supporting early warning systems for waterborne outbreaks (Table 1).

**Table 1:** Different biosensors with transducers, chip types, LODs^s^, and linear/dynamic ranges for the detection of *Cryptosporidium* spp.

Analyte	Type of sensor	Transducer	Type of chip (electrode)	LOD	Linear/dynamic range	Source	Reference
*C. parvum*	Immunosensor	Electrochemical sensor	Screen-printed carbon electrodes	2 × 10^4^ oocysts mL^−1^	Not reported	Ground beef	(Nze et al., 2019)
*C. parvum*	Immunosensor	Optical microfluidic biosensor	Microfluidic chip based on an immune agglutination assay	10^0^ to 10^1^ oocysts mL^−1^	10^0^ to 10^4^ oocysts mL^−1^	Water sample	(Angus et al., 2012b)
*C. parvum*	Immunosensor	Optical (colorimetric, enzymatic amplification + AuNP signal enhancement)	Not reported (nitrocellulose membrane)	10 oocysts mL^−1^	3 to 200 oocysts mL^−1^	Water samples	(Thiruppathiraja et al., 2011a)
*Cryptosporidium* spp.	Immunosensor	Sandwich enzyme-linked electrochemical biosensor	Indium Tin Oxide (ITO) electrode	3 oocysts mL^−1^	5–100 oocysts mL^−1^	Water samples	(Thiruppathiraja et al., 2011b)
*C. parvum*	Immunosensor	Optical SPR biosensor	Gold-coated, polyclonal anti-mouse IgM arrayed SPR sensor chip	1 × 10^2^ oocysts mL^−1^	1 × 10^6^ to 1 × 10^2^ oocysts mL^−1^	Natural water systems	(Kang et al., 2008)
*Cryptosporidium* spp.	Immunosensor (labeled)	Evanescent wave fiber (optic chemical sensor)	R Plus 4S^b^	10^5^ oocysts mL^−1^	10^6^ oocysts mL^−1^	Water sample	(Kramer et al., 2007)
*C. parvum*	Immunosensor	Piezoelectric electrochemical sensor	Quartz crystal microbalance	3 × 10^5^ to 10^7^ oocysts mL^−1^	3 × 10^5^ to 1 × 10^7^ oocysts mL^−1^	Water sample	(Poitras et al., 2009)
*C. parvum*	Immunosensor	EIS, CV, SWV	Microfabricated; thiolated gold electrodes	20 oocysts /5 μL	0 to 300 oocysts	Water sample	(Luka et al., 2022)
*C. parvum*	Immunosensor	CV	Interdigitated gold electrodes	40 cells/mm^2^	15 to 153 cells/mm^2^	Water sample	(Luka et al., 2019)
*C. parvum*	Immunosensor	EIS	Interdigitated micro-electrode	10 cells μL^−1^	1.43433 × 10^−5^ × C + 7.545921 × 10^−4^	Water sample	(Houssin et al., 2010)
*C. parvum*	Immunosensor	Piezoelectric-excited millimeter-sized cantilever (PEMC^n^)	PZT^e^ and glass film	10^2^, 10^3^, and 10^4^ oocysts mL^−1^	10^2^ to 10^4^ oocysts mL^−1^	Drinking water	(Campbell and Mutharasan, 2008)
*C. parvum*	Immunosensor	Colorimetric detection (non-labeling fluorescence sensor)	Polydiacetylene-based fluorescence chip	1 × 10^3^ oocysts mL^−1^	1 × 10^2^ to 1 × 10^5^ oocysts mL^−1^	Water sample	(Park et al., 2008)
*C. parvum*	Immunosensor (FITC-labeled antibody-based)	Optical (Fluorescence detection via microscope)	PDMS^m^ microfluidic device integrated with a SUS^i^ micromesh	10 oocysts mL^−1^	18–200 oocysts mL^−1^	Contaminated water	(Taguchi et al., 2007)
*C. parvum*	Immunosensor	Optical SPR biosensor	Gold chip	1 × 10^6^ oocysts mL^−1^	Not reported	Oocyst Stock Waterborne, Inc. (USA)	(Kang et al., 2006)
*C. parvum*	CRISPR/Cas12a-powered immunosensor	Fluorescence plate reader	Not reported (streptavidin-coated 96-well plate with biotinylated antibody capture interface)	Single oocyst per sample (∼10 oocysts mL^−1^)	6.25 to 1600 oocysts mL^−1^	Water sample	(Li et al., 2021)
*C. parvum hsp70 mRNA*	Genosensor	Optical/colorimetric transducer	PMMA microfluidic chip with surface modifications	30 oocysts	Not reported	Commercial oocysts (Waterborne Inc.)	(Reinholt et al., 2014)
*Cryptosporidium* spp.	Genosensor	Optical; colorimetric (camera for signal transmission)	Micro-fabricated chip; thiolated oligonucleotides/AuNPs (sensing probe)	5.0 μM	5 to 100 μM	Water (*Cryptosporidium* RNA)	(Luka et al., 2021)
*Cryptosporidium* spp. DNA	Genosensor	Electrochemical DPV^d^ and EIS	3D μTAS^f^	1.8 ng mL^−1^	2.5 ng mL^−1^ - 0.1 μg mL^−1^	*Cryptosporidium* DNA	(Ilkhani et al., 2019)
*C. parvum*	Genosensor	Amperometric electrochemical biosensor	IDUA integrated with a gold electrode	1 oocyst mL^−1^	Not reported	*C. parvum* oocyst DNA	(Nugen et al., 2009)
*C. parvum*	Solution-phase genosensor	Nanoparticle-based optical sensor	AuNP Probes	670 oocysts μL^−1^ reaction mix	4 × 10^5^ and 4 × 10^6^ copies of RNA μL^−1^	Stool sample (100 mg)	(Weigum et al., 2013)
*C. parvum, G. duodenalis, E. coli,* and impurities	Optical sensor	Optical (CMOS^j^ camera; PMT^k^ for triggering)	Glass microfluidic chip	Not reported	3000 parasites/min at a flow rate of 12 μL/min (accuracy >96%)	Contaminated water	(Huang et al., 2017)
*C. parvum*	Optical SPR biosensor	Plasmonic multilayer structure	Prism-coupled SPR chip with Ag, TiO₂, and MXene layers	3 × 10^−5^ RIU^a^	Not reported	Contaminated water	(Daher et al., 2025)
*C. parvum*	Optical sensor using DIHM*	650 nm laser and CMOS* camera	Not reported (3.5 mL optical glass cuvette)	0.33 oocysts mL^−1^	0.3 to 300 oocysts mL^−1^	Lab-cultured Iowa isolate oocysts	(Thompson et al., 2020)
*C. parvum, G. duodenalis*	Label-free optical biosensor (100% accuracy)	Optical (DPM^l^ system with a CCD camera)	PDMS^m^ microfluidic chip	Not reported	Not reported	Drinking water	(Gu et al., 2019)
*C. parvum*	Electrochemical aptasensor	SWV with GNP-SPCE^⁎^	Not reported (SPCE modified with gold nanoparticles)	∼10^3^ oocystsmL^−1^	50 to 900 oocysts	Drinking and recreational water	(Iqbal et al., 2019)
*C. parvum*	Electrical impedance biosensor	IMAs^o^ functionalized with HCT-8 cells	Gold-coated Pyrex substrate with IMAs^o^	1 × 10^2^ oocysts mL^−1^	1 × 10^2^ to 1 × 10^6^ oocysts mL^−1^	Oocysts from Waterborne Inc. (New Orleans, LA, USA), Iowa strain	(Dibao-Dina et al., 2015)
*C. parvum*	Aptamer	CV, EIS, chronocoulometry	3D gold NMI*	10^3^ to 10^4^ oocysts mL^−1^	10^1^ to 10^5^ oocysts mL^−1^	Oocysts retrieved from buffer, tap water, and stool	(Siavash Moakhar et al., 2023)
*Cryptosporidium* spp.	Aptamer-conjugated magnetic beads	Not reported (flow cytometry and fluorescence microplate-based assay)	Not reported (uses micro-magnetic beads)	5 oocysts/300 μL	Not reported	Waste sample	(Hassan et al., 2021a)
*C. parvum*	Aptasensor	SWV	Screen-printed carbon electrode	100 oocysts mL^−1^	200 to 700 oocysts mL^−1^	Fresh fruits	(Iqbal et al., 2015)
*C. parvum*	Antibodies and aptamers	Colorimetric/ fluorescence detection	Not reported (syringe filters)	1 × 10^5^ oocysts mL^−1^	1 × 10^3^ to 1 × 10^8^ oocysts mL^−1^	Recreational water	(Ruiz-andino, 2022)
*Cryptosporidium* spp.	IMS combined LAMP^h^-based detection	Fluorescence and colorimetric detection	Magnetic beads functionalized with anti-*Cryptosporidium* monoclonal antibodies for target capture)	5 to 10 oocysts per 10 mL	5 to 1000 oocysts per 10 mL	Commercial oocysts (from Biopoint Pty. Ltd., Sydney)	(Mahmudunnabi et al., 2025)
*C. parvum*	CIP^p^ biosensor	Fluorescence microscopy (staining with the Crypt-a-Glo antibody kit and DAPI^r^ for oocyst visualization).	PDMS^m^ surfaces	100 oocysts mL^−1^	100 to 100,000 oocysts mL^−1^	Lab-cultured Iowa isolate	(Sarkhosh et al., 2021)

RIU^a^: Refractive index unit; R Plus 4S^b^: RAPTOR Plus 4S; NMI^c^: Nano−/microisland; DPV^d^: Differential pulse voltammetry 0.0375 to 1.2 AU mL^−1^and 2.0 to 18 AU mL^−1^, PZT^e^: Lead Zirconate Titanate; 3D μTAS^f^: Three-dimensional micro total analysis systems; GNP-SPCE^g^: Gold nanoparticle-modified screen-printed carbon electrode; LAMP^h^: Loop-mediated isothermal amplification-based detection; SUS^i^: Special use stainless 304; CMOS^j^: Complementary metal-oxide-semiconductor; PMT^k^: Photomultiplier tube; DPM^l^: Diffraction phase microscopy; PDMS^m^: Polydimethylsiloxane; PEMC^n^: Piezoelectric-excited millimeter-sized cantilever; IMAs^o^: Interdigitated Microelectrode Arrays; CIP^p^: Cell-imprinted polymer; DIHM^q^: Digital inline holographic microscopy; DAPI^r^: 4′,6-diamidino-2-phenylindole. Note: LODs represent analytical sensitivities determined primarily under optimized laboratory conditions using spiked samples (typically 5–100 μL volumes).

##### 5.2.1.2 Label-free affinity capacitive biosensor

Capacitive biosensors guarantee label-free, ultrasensitive detection of biomarkers by measuring capacitive variations from biomarker-bioreceptor interactions. Consisting of bioreceptors and transducers, the dielectric layer changes due to binding events, translating into measurable signals. Owing to their high sensitivity and low cost, these are ideally used for food safety monitoring. The transducers employ gold (AuEs), carbon (CEs), or interdigitated electrodes (IDEs) to immobilize the bioreceptor and generate a detectable capacitive signal (Huang et al., 2024).

Luka et al. (2019) developed a label-free capacitive biosensor using interdigitated gold electrodes to detect *Cryptosporidium* oocysts in water. The platform incorporated a capture probe, anti-*Cryptosporidium* mAbs (IgG3), and bovine serum albumin (BSA) to enhance specificity (Table 1) (Luka et al., 2019). *Cryptosporidium* detection workflow included coating the sensing electrodes with anti-*Cryptosporidium* antibodies and incubation with water samples containing varying oocyst concentrations; washing with phosphate-buffered saline (PBS, pH 7.0) to remove unbound or nonspecific molecules; drying the IDEs under controlled conditions; and capacitance measurements performed across a specific frequency range to quantify oocyst binding. Future work needs to be focused on electrode material, immobilization techniques, and signal implications to enhance their performance.

##### 5.2.1.3 Electrochemical aptasensor

**Fig. 6:**
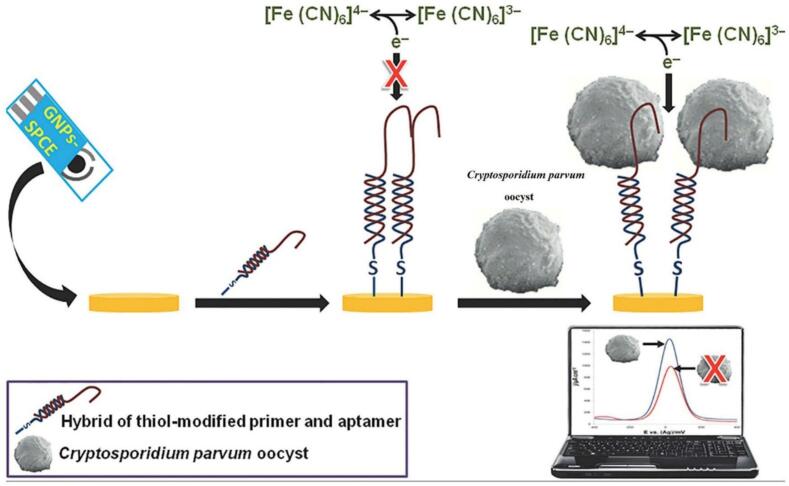
Schematic of an electrochemical aptasensor for *C. parvum* oocysts detection using screen-printed electrodes. The biosensor employs a thiol-modified aptamer-primer hybrid immobilized on the electrode surface. The binding of *C. parvum* oocysts disrupts electron transfer between [Fe (CN)₆]^3−^/^4−^ redox probe and the electrode, detectable via voltammetry. This system leverages aptamer specificity to enable label-free oocyst identification, with signal inhibition correlating to target concentration. Adapted from (Iqbal et al., 2015), PLoS ONE 10(9): e0137455 (https://doi.org/10.1371/journal.pone.0137455). Licensed under CC BY 4.0 (https://creativecommons.org/licenses/by/4.0/).

Aptamers are synthetic single-stranded DNA/RNA oligonucleotides, designed to bind specific target molecules with high affinity. Aptamers are commonly selected by systematic evolution of ligands by exponential enrichment (SELEX). Aptamers are frequently used in diagnostics and biosensors, including for *Cryptosporidium* spp. identification, due to their higher stability, high affinity, longer shelf lives, and lower costs of production than antibodies (Liu et al., 2020). In this context, Iqbal et al. (2015) developed a first electrochemical nanomaterial-based aptasensor using a screen-printed carbon electrode (SPCE) functionalized with AuNPs to detect *C. parvum* oocysts in fruit samples. Their design used 14 aptamer variants and thiolated DNA primers to immobilize the hybrid complex on SPCE. This aptasensor obtained a reasonable LOD and dynamic range (Table 1), facilitating food safety surveillance (Fig. 6) (Iqbal et al., 2015). However, reliance on AuNPs to enhance sensitivity increases fabrication cost and complexity, while the limited commercial availability of aptamers poses additional challenges. Addressing these challenges in the future can lead to the development of better practical aptamers for on-site detection of PPs.

Iqbal et al. (2019) expanded their work on spiked water samples by incorporating an AuNP-modified SPCE into an electrochemical aptasensor, where a biotinylated aptamer was bound to streptavidin-coated magnetic beads. Square wave voltammetry was used to monitor the increase in current, thereby improving the LOD to 50 oocysts. Aptamer-based biosensors are promising analytical tools for pathogen detection, including oocyst identification. However, there are certain challenges in their applications. The SELEX aptasensor selection method involves screening large oligonucleotide libraries through multiple rounds of selection and amplification. In addition, the variable and complex structure of oocysts can affect aptamer performance, highlighting the need to enhance SELEX efficiency to achieve high specificity for whole-cell or protozoan targets (Siwak et al., 2023).

##### 5.2.1.4 Magneto-electrochemical biosensor

Simple electrochemical sensors are constrained by complex immobilization protocols, low repeatability, poor stability, and insufficient strategies for ultrasensitive detection. To address these limitations, magnetic nanoparticles (NPs), particularly iron oxide NPs exhibiting superparamagnetic properties and widely employed in magnetic separation (Chang et al., 2022; Wu et al., 2015), have been integrated, giving rise to magneto-electrochemical biosensors. These biosensors address the issues associated with simple electrochemical biosensors by efficiently isolating the target from complex matrices using external magnets, enabling electrode regeneration, and boosting detection limits due to integration with enzymes, DNA-based technologies, and nanomaterials. Their working principle includes the recognition phase, separation/preconcentration, signal generation, and quantifying the redox current, correlating with target concentration (Chang et al., 2022).

For example, immunomagnetic separation (IMS) effectively isolates *Cryptosporidium* spp. and *G. duodenalis* oocysts/cysts from water samples ranging from 10 L (https://bit.ly/3Zk3D83). In this context, Nze et al. (2019) devised an electrochemical detection protocol for *C. parvum* in ground meat, utilizing immunomagnetic capture after hydrodynamic cavitation pretreatment, followed by cavitation of meat samples. Immunomagnetic capture of the pathogens was employed to eliminate non-target molecules, followed by signal amplification via polyG-coated secondary beads. The redox activity of the polyG label was subsequently monitored via SWV, enabling indirect quantification of *C. parvum* isolated from meat. This work streamlined sample preparation and detection to develop an integrated and fully automated platform to precisely recognize and quantify the multiple food- and waterborne pathogens (Nze et al., 2019).

Developing high-quality nanoparticles, combining magneto-biosensors with microfluidics for high-throughput, POC detection, advancing signal amplification through the use of CRISPR/Cas system, and optimization with DNA walkers and catalytic hairpin assembly for rapid protein detection, through a multidisciplinary approach, will enhance the field's tractability (Chang et al., 2022).

##### 5.2.1.5 Other types of electrochemical biosensors

Previously, different types of electrochemical biosensors have advanced *Cryptosporidium* detection. Nugen et al. (2009) developed a polymethyl methacrylate (PMMA) microfluidic electrochemical biosensor using magnetic beads-liposome complex and interdigitated ultramicroelectrode array (IDUA), achieving sensitivity down to one oocyst (Table 1), but faced cost and complexity limitations (Nugen et al., 2009). In contrast, the potentiometric biosensor developed by Laczka et al. (2013) utilized horseradish peroxidase (HRP)-labeled secondary antibodies, improving the LOD by 100–1000-fold compared to ELISA, without requiring expensive antibodies (Table 1) (Laczka et al., 2013). Alternatively, Houssin et al. (2010) demonstrated an EIS-based (label-free) biochip with an interdigitated microelectrode array to detect *Cryptosporidium* spp. in water samples (Table 1), offering real-time discrimination of viability via impedance shift between 10 kHz and 100 kHz. These innovations highlight trade-offs between sensitivity, cost, and practicality, with the amperometry system excelling in sensitivity, potentiometry in affordability, and EIS in label-free viability analysis (Houssin et al., 2010).

#### 5.2.2 Optical biosensors

##### 5.2.2.1 Fluorescence-based, SPR and microfluidic-integrated optical biosensor

Optical biosensors integrate bioreceptors with optical transducers to enable the detection of output after interacting with analytes (Daramola et al., 2025). The signal intensity is proportional to the analyte concentration. The underlying mechanism is based on measuring the amount of absorbance, fluorescence, luminescence, and change in color and appearance or disappearance of color (Daramola et al., 2025). Optical biosensors provide appropriate equipment that uses SPR, absorption, fluorescence, Raman scattering, and reflectance to detect multiple kinds of analytes (Daramola et al., 2025). Fluorescence-based optical biosensors (FOBs) detect foodborne pathogens using antibodies to capture the target pathogen in a sample (Daramola et al., 2025). In this context, Kramer et al. (2007) devised a sandwich immunoassay for *Cryptosporidium* oocysts, combining anti-oocyst monoclonal antibodies for capture with cyanine 5 (Cy5)-labeled polyclonal antibodies for detection. Sensitivity was enhanced tenfold by specimen heating and by employing the polyclonal detection antibody (Kramer et al., 2007). To address fluorescence's short, excited-state lifetime, Gaft et al. (2015) introduced a luminescence-based mechanism enabling photon emission at any time (Gaft et al., 2015).

Additionally, to improve the sensitivity of optical biosensors, SPR biosensors based on the refractive index were created (Zeng et al., 2021). SPR biosensors, leveraging refractive index changes, enhance optical biosensor sensitivity through label-free, real-time analysis with high specificity and throughput capacity, making it appropriate for food quality monitoring and ecological applications (Olaru et al., 2015; Zeng et al., 2021). In this regard, Kang et al. (2006) designed a flow-type SPR biosensor for rapid *Cryptosporidium* oocysts detection using mixed self-assembled monolayers (SAMs) (11-mercaptoundecanoic acid (11-MUA) and 3-mercaptopropanol (3-MPOH)). Nemati et al. (2023) noted that the biotin–streptavidin chemistry employed in this system enhances analyte accessibility to the sensor surface, improving the detection limit from 1 × 10^6^ to ∼1 × 10^2^ oocysts/mL (Kang et al., 2006) (Table 1).

Microfluidic chips, as micro-total analysis systems, provide ideal platforms for integrating high-throughput optical biosensors that enable simultaneous detection of multiple pathogens in a single assay (Liao et al., 2019). Merging the versatility of optical sensing with the precision of microfluidics yields powerful tools for rapid, portable biosensing; for example, Angus et al. (2012a) demonstrated a field-deployable optical microfluidic biosensor capable of near-real-time, single-oocyst-level detection of *C. parvum* in field water samples. The system consists of a dual-inlet, single-outlet microfluidic design but requires labels (e.g., microbeads) and prior antigen removal from the oocyst to enable immunoagglutination. Despite the sensitivity (sub-single-oocyst detection with approximately 15 mL sample injection of the established sensing platform), the system necessitates labels like microbeads; on-site application is limited by these prerequisites (Angus et al., 2012a). When paired with filtration/concentration, this method can detect and identify ≤1 oocyst in large water volumes, matching or exceeding US-EPA 1623.1 efficiency. It reduces time and labor compared to staining microscopy with a one-minute detection assay (following a 4-min sonication step), and a linear range spanning five orders of magnitude. However, without filtration, the LOD was 1–10 oocysts mL^−1^, restricting its utility for rapid, near real-time field testing (Angus et al., 2012a).

Raman spectroscopy is a light scattering technique that utilizes monochromatic light to excite molecules to higher energy states, leading to emission of radiation at different wavelengths, a phenomenon called Raman scattering. As the Raman signals are typically weak, coating the sensing surface with noble metals enhances the signal through Surface-Enhanced Raman Spectroscopy (SERRS). In this context, Rule and Vikesland (2009) developed a SERRS biosensor for the simultaneous detection of *C. parvum* and *Giardia duodenalis* (using immunogold labels-AuNPs linked to monoclonal antibodies tagged with rhodamine B isothiocyanate and malachite green isothiocyanate, respectively (Rule and Vikesland, 2009). These sensors demonstrate high sensitivity and specificity without antibody cross-reactivity, highlighting the potential of Raman-based multiplex pathogen detection. Despite these advances, SERRS biosensors face challenges in *Cryptosporidium* spp. detection, including difficulties in achieving reproducibility and quantitative results, with no studies reporting a precise detection limit (Luka et al., 2022). The time of acquisition is lengthy, up to 15–20 min per oocyst, and only samples with high concentration and low volume can be analyzed efficiently. Additionally, oocysts must be immobilized on the sensing surface, as their movement may interfere with measurements (Siwak et al., 2023).

#### 5.2.3 Colorimetric biosensor

**Fig. 7:**
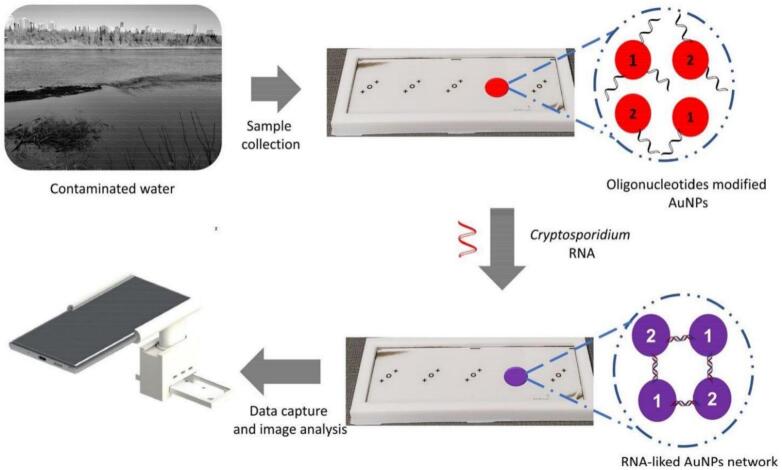
Schematic of label-free *Cryptosporidium* RNA biosensor for contaminated water analysis. Sample collection proceeds with AuNPs functionalized with *Cryptosporidium*-specific oligonucleotides. Target RNA hybridization induces AuNPs' aggregation and a colorimetric shift (absent with non-complementary RNA), analyzed via smartphone imaging, enabling portable pathogen detection. Adapted from (Luka et al., 2021), Sci Rep 11:23192. (https://doi.org/10.1038/s41598-021-02580-w) Licensed under CC BY 4.0 (https://creativecommons.org/licenses/by/4.0/).

Colorimetric biosensors rely on identifying the color changes produced by biorecognition events. These color shifts can be measured optically in proportion to the analyte or assessed visually (Luka et al., 2015)*.* Connelly et al. (2008) developed a microfluidic strip-based colorimetric biosensor capable of detecting a single *Cryptosporidium* oocyst in a 10 μL sample using a flow cytometer. However, the system encountered challenges from sample contaminants, microbes, and other limitations. Furthermore, Luka et al. (2021) developed a portable, colorimetric biosensor for *Cryptosporidium* RNA using oligonucleotide-functionalized AuNPs, enabling highly sensitive, specific, and label-free detection. Thiolated oligonucleotides complementary to adjacent *Cryptosporidium* RNA sequences were immobilized on AuNPs. Hybridization with target RNA caused nanoparticle aggregation, resulting in a noticeable color shift visible to the naked eye, while non-complementary RNA caused no color change. The test was built into a microfluidic chip housed in a 3D-printed device, eliminating the need for expensive lab equipment. A mobile camera records color variation for quantitative investigation. The linear range and the LOD for *Cryptosporidium* RNA are given in Table 1. This compact, affordable biosensor offers rapid, user-friendly pathogen monitoring in resource-limited settings, effectively identifying multiple *Cryptosporidium* spp. in water samples (Fig. 7) (Luka et al., 2021).

#### 5.2.4 CRISPR/Cas12a-based fluorescent lateral flow strip (LFS) biosensor

Innovations in the CRISPR/Cas12a-based biosensing have resulted in powerful, fast, and highly specific on-site detection tools for parasite identification. The basic principle of this technology involves amplification of the target sequence using recombinase polymerase amplification (RPA), without demanding thermal cycling, making it better suited for POC use. Design and preparation of crRNA, Cas12a/crRNA complex formation, target recognition, trans-cleavage of reporter molecules, followed by signal detection through fluorescence or LFS, constitute the primary working principle of such platforms for the detection of foodborne pathogens (Yu et al., 2021). This approach makes them suitable for resource-limited areas.

The recent integration of the CRISPR/Cas12a system into a fluorescent lateral flow strip biosensor for on-site detection of *C. parvum* subtype family IId demonstrates the viability of CRISPR/Cas-based parasite detection. Utilizing the Cas12a/crRNA trans-cleavage mechanism, this system identifies as little as a single copy of the cloned *C. parvum* GP60 gene and 10 OPG following simplified sample preparation involving brief heat treatment with N-lauroylsarcosine sodium salt, eliminating complex commercial DNA extraction procedure or multi-step purification protocols. The assay exhibits no cross-reactivity with other *C. parvum* subtype families or common enteric protozoa. This subtype-specific detection is particularly valuable for food and water safety, as the IId subtype is the predominant zoonotic *C. parvum* strain for human-ruminant transmission. The method enables rapid source tracking during outbreaks, epidemiological surveillance of contaminated supplies, and public health interventions without requiring trained operators or specialized equipment (Yu et al., 2021).

The combined RPA and CRISPR/Cas12a (ReCTC) detection assay involves the *C. parvum* chromosome 6, incorporating primer positions, target DNA sequence, crRNA binding site, and protospacer adjacent motif (PAM). It introduces RPA-amplified DNA into the system, where target recognition initiates ternary complex formation and enables visual quantification of analyte concentrations. The components include a fluorescent reporter, a quencher, and biotin, which enable fluorescence-based signal identification (Yu et al., 2021).

Table 1 comprehensively presents different types of biosensors for detecting *Cryptosporidium* spp., underscoring the advancements regarding multiplexing, sensitivity, and portability. Recent innovations involve the 3D nano(gold)-structured microelectrode-based genosensor (Siavash Moakhar et al., 2023) to quantify lower concentrations of oocysts in water samples, hence, the development of label-free optical biosensors (Gu et al., 2019). Emerging technologies such as EIS microarrays (Luka et al., 2022) and aptamer-based conjugated magnetic-based platforms exhibit increased specificity and field adaptability. These platforms expand electrochemical, optical, and genosensor modalities, with trends toward simplified workflows (e.g., microfluidics, lyophilized reagents) and integration with nanomaterials, e.g., AuNPs, silver nanoparticles, to improve LOD and real global applicability for environmental and clinical monitoring.

### 5.3 Types of biosensors aimed at detecting *T. gondii* in food and water samples

#### 5.3.1 Electrochemical biosensors

Following the successful application of electrochemical biosensors for *Cryptosporidium* spp., they have increasingly been adapted for *T. gondii*. Their appeal lies in their straightforward fabrication, requirement for small sample volumes, and ability to produce measurable signals from molecular interactions on electrode surfaces (Nemati et al., 2023).

##### 5.3.1.1 DNA-based label-free electrochemical biosensors

As a kind of excellent biomaterial, DNAs have been integrated to build various biosensors via interaction between biomolecules and DNAs, because of the advantages linked to DNA-based biosensors, such as rapid detection and operational comfort, in addition to high sensitivity and specificity (Hai et al., 2020). However, DNA-based biosensors mostly suffer from labeling requirements for nanomaterials or electroactive molecules on DNA as signal readout elements, which complicate operation and increase the cost of production. Label-free DNA-based biosensors appear as an excellent alternative in this regard; they are based on the intrinsic properties of DNA (aptamer binding, DNA hybridization, or catalytic activity) to transduce target recognition proportional to electrochemical signals. Nanomaterials and amplification strategies positively influence sensitivity, enabling ultra-low concentrations of targets (proteins, nucleic acids, and small molecules) (Hai et al., 2020).

Gökce et al. (2016) developed a single-use electrochemical DNA biosensor for the voltammetric detection of sequence-specific DNA hybridization related to *T. gondii*. This label-free method detects DNA duplex formation by monitoring the guanine oxidation signal generated during DNA hybridization. The design of the biosensor consists of immobilizing a guanine-free, inosine-modified probe on the pencil graphite electrode (PGE) through the process of wet adsorption. The hybridization between the probe and target DNA was assessed using differential pulse voltammetry (DPV), with the guanine oxidation signal detected at +1.0 V on the PGE surface. Optimization studies revealed maximum DNA hybridization efficiency at a target concentration of 40 mg mL^−1^ and a hybridization time of over 50 min. Specificity tests demonstrated the biosensor's selectivity against non-complementary and mismatched DNA sequences. Under ideal conditions, the guanine oxidation signal indicating complete hybridization was identified at target concentrations shown in Table 2. The system efficiently recognized *T. gondii* DNA hybridization in PCR amplicons, indicating its potential in volumetric testing (Gökce et al., 2016).

**Table 2:** Different biosensors with transducers, chip types, LODs, and linear/dynamic ranges for the detection of *T. gondii.*

Analyte	Type of sensor	Transducer	Type of chip	LOD	Linear/dynamic range	Source	Reference
*T. gondii*	Immunosensor	Electrochemical biosensor (label-free/DPV)	Pencil graphite electrode	1.78 μg mL^−1^	0.5 to 25 μg mL^−1^	DNA sample	(Gökce et al., 2016)
*T. gondii*	Immunosensor	Optical	Human recombinant galectin-3 and galectin-1 coupled to CM5 sensor chip (carboxymethylated dextran surface)	Not reported	0.2 to 1 μM	GPIs	(Debierre-Grockiego et al., 2010)
*T. gondii*	Immunosensor	Optical SPR biosensor	Not reported (azurin, azurin-like protein (Laz) with SAG1)	Not reported	0.1 to 10 mM	SAG1	(Naguleswaran et al., 2008)
*Toxoplasma* spp.	Immunosensor	Piezoelectric	Interaction of rTgMIC4 with glycoproteins utilized QCM-D^a^	Not reported	Not reported	rTgMIC4	(Santos et al., 2015)
*T. gondii*	Immunosensor	Microfluidic optical biosensor	Microfluidic chip	87 fg mL^−1^	1 pg mL^−1^ to 10 ng mL^−1^	Lab-cultured oocysts	(Chen et al., 2023)
*T. gondii, Cryptosporidium* spp., and *G. duodenalis*	Genosensor	Optical	Not reported (CHAS)	Not reported	0.5 mg mL^−1^ to 20 μg mL^−1^	DNA from contaminated fresh lettuce, parsley, and water samples	(Reiling et al., 2021)
*T. gondii*	Genosensor	Electrochemical biosensor/DPV	Graphite electrodes; AuNPs	100 ng μL^−1^	100 to 400 ng μL^−1^	DNA samples	(Alves et al., 2017)
*T. gondii*	Genosensor	Magnetic fluorescent nanoparticles (Fe3O4/CdTe)	Solution-based assay (no physical chip used)	8.339 nM	Not reported	DNA oligonucleotides	(He et al., 2015)
*T. gondii*	Genosensor	Magnetic-fluorescent CdTe @Ni quantum dots (mQDs)	Not reported	2.70 × 10^−9^ mol L^−1^	Not reported	DNA	(Xu et al., 2013)
*T. gondii*	Genosensor (FRET-based)	Fluorescence signal (CdTe/Fe₃O₄ quantum dots as donor and BHQ-2b as quencher)	Not reported (magnetic nanoparticle-based system)	Not reported	Not reported	Commercial DNA oligonucleotides (S1, S2, S3)	(Liang et al., 2009)
*T. gondii*	Genosensor (colorimetric)	BTB	No chip (conventional tubes)	0.014 *T. gondii* μL^−1^	Not reported	ab-cultured stool samples DNA	(Zhang et al., 2022)
*T. gondii*	Colorimetric biosensor	BTB	Not specified	As low as 0.014 *T. gondii* μL^−1^	Not reported	Lab-prepared fecal sample DNA	(Wang et al., 2024)

QCM-D^a^: Quartz crystal microbalance with dissipation monitoring; BHQ-2^b^: Black hole quencher-2.

Additionally, the integration of electrochemical techniques with nanomaterials (1 to 100 nm) like graphene, carbon, metallic nanotubes, organic polymers, silica nanoparticles, nanowires, and indium tin oxide (ITO) enhances biosensor performance and enables scalable production due to their high electrical conductivity (Singh et al., 2021). In this context, Alves et al. (2017) developed an electrochemical biosensor to detect *T. gondii* DNA, using a graphite carbon electrode modified with poly 3-hydroxybenzoic acid and functionalized with the ToxG1 probe. The sensor employed DPV to measure guanine oxidation signals triggered by hybridization with *T. gondii* genomic DNA, distinguishing complementary from non-complementary targets, with a detection limit of *T. gondii* DNA (Table 2). Hybridization between the ToxG1 probe and *T. gondii* DNA was further validated using optical assays with AuNPs, which exhibited a distinct shift in the probe's absorbance spectrum upon target binding (Alves et al., 2017).

#### 5.3.2 Optical biosensors

##### 5.3.2.1 *T. gondii* autophagy-related proteins and SPR biosensor

Autophagy, controlled by autophagy-related proteins (Atg), is an important intracellular degradation mechanism in eukaryotes such as *T. gondii* that is required for cellular homeostasis and survival. This mechanism is based on ubiquitin-like conjugation systems, specifically Atg8-phosphatidylethanolamine (PE) and Atg5-Atg12 (Subauste, 2019). In a study by Chen et al. (2016), the conditional knockdown of *T. gondii* Atg3 (TgAtg3) genes disrupted TGAtg8 lipidation to PE, impairing parasite survival. The interaction between TgAtg8 and TgAtg3 is vital for *T. gondii* survival, positioning it as a potential target for parasite identification using SPR biosensing techniques. To investigate this interaction, the researchers immobilized pure TgAtg3 on a biosensor chip and activated it with 10 mM NiSO₄. SPR study with different doses of TgAtg8 indicated a significant binding affinity at 34.9 nM TgAtg8 (Chen et al., 2016). These findings provided a foundation for targeting the TgAtg8/TgAtg3 interaction in food surveillance systems. Using nanoparticles may improve the sensitivity and precision of SPR-based identification for this type of system.

##### 5.3.2.2 Colorimetric-biosensor-based detection of DNA hybridization

As previously stated, microscopy requires a high level of knowledge and expertise and is less sensitive than colorimetric-biosensor-based detection of DNA. These types of biosensors detect the target DNA sequences through a visible color change, enabling rapid detection without the necessity of complex instruments. Their working mechanism involves DNA probes that hybridize with the DNA of interest, leading to variations in nanoparticle aggregation or enzymatic activity that generate a colorimetric signal corresponding to the hybridization event, leading to real-time detection (Syed et al., 2024; Xia et al., 2010).

In this context, the study by Reiling et al. (2021) describes a cloth-based hybridization array system (CHAS) as an alternative to Sanger sequencing for confirming PCR-positive specimens. CHAS is a low-cost, fast, and efficient technique for the simultaneous detection of multiple protozoan parasite species through the colorimetric identification of PCR amplicons on a polyester cloth. PCR primers and CHAS hybridization probes were developed for the detection of *Cryptosporidium* spp., *T. gondii,* and *G. duodenalis*. When tested on artificially contaminated fresh produce (lettuce, parsley) and water samples (river and wastewater), this CHAS assay successfully detected *Cryptosporidium* spp., *T. gondii,* and *G. duodenalis*. This study demonstrated that the CHAS detection method provided a simple and cost-effective alternative to DNA sequencing for confirming PCR-positive results in laboratories that test food and water samples for parasites (Reiling et al., 2021). This assay may prove particularly beneficial in developing countries, where access to DNA sequencing facilities is often limited (Table 2).

##### 5.3.2.3 RPA-CRISPR/Cas12a-based LFS biosensor

As discussed for *Cryptosporidium* spp., Lei et al. (2022) developed a portable, on-site detection system for *T. gondii* by integrating RPA with the CRISPR/Cas12a platform. This technique uses a glass microfiber filter to extract *T. gondii* DNA from low-concentration samples. Lyophilized RPA and Cas12a/crRNA reagents are preloaded into a single Eppendorf tube, enabling reactions to be conducted at 37 °C using a low-cost thermal controller. Targeting the *T. gondii* B1 gene, the system achieves a detection limit of 3.3 copies μL^−1^ with high specificity. Sample enrichment and lysis are followed by RPA and CRISPR/Cas12a detection, with fluorescence-based readout enabling portable, rapid (< 35 min), sensitive, and on-site analysis. The system, validated by real-time fluorescence intensity measurement (0–35 min), offers flexibility for field deployment, with results visualized using a fluorometer or LFS. This resilient and quick platform is ideal for on-site detection in remote areas (Lei et al., 2022), and shows promise for detecting *T. gondii* in environmental and food products.

Table 2 highlights innovative biosensor-based approaches for *T. gondii* detection in food, water, and DNA-based samples. Debierre-Grockiego et al. (2010) designed an SPR biosensor targeting the interaction between glycosylphosphatidylinositols (GPIs) and gelatin-3, underscoring its role in the recognition of macrophages, which could inform diagnostic and therapeutic strategies. Naguleswaran et al. (2008) used SPR to demonstrate binding of an azurin-like protein to *T. gondii* surface antigen 1 (SAG1), thereby highlighting a novel inhibition mechanism for parasite invasion. Similarly, Santos et al. (2015) utilized a quartz crystal microbalance with dissipation monitoring (QCM) to elucidate the interaction between recombinant *T. gondii* micronemal protein 4 (rTgMIC4) and glycoproteins, thereby clarifying host-pathogen adhesion. Liang et al. (2009) and He et al. (2015) explored fluorescence energy transfer (FRET) using CdTe/Fe_3_O_4_ quantum dots and magnetic fluorescent particles, respectively, achieving sensitive *T. gondii* DNA identification with low LOD. Xu et al. (2013) devised CdTe@Ni quantum dots for magnetic-fluorescence DNA sensing, exhibiting a LOD of 2.70 × 10^−9^ mol L^−1^. Wang et al. (2024) introduced a bromothymol blue (BTB)-based colorimetric biosensor for the visual detection of *T. gondii* DNA in fecal samples, with minimal instrumentation. Zhang et al. (2022) developed a stem-loop primer-assisted isothermal amplification platform for semi-quantitative detection of *T. gondii,* synchronizing simplicity with visual readouts (Table 2). These studies collectively advance biosensor versatility, streamline workflows, and enhance field applications for parasitic detection.

## 6 Transitioning biosensor applications: from clinical diagnosis to foodborne detection of PPs for early outbreak prevention

Biosensors for *Cryptosporidium* spp. and *T. gondii* primarily utilize immunoassays and genosensors for clinical diagnosis. For *Cryptosporidium* spp., biosensors have been validated in lab-cultured oocysts (Thompson et al., 2020), stool (Laczka et al., 2013), and DNA samples (Ilkhani et al., 2019). For *T. gondii*, biosensors have been developed for serum (Jiang et al., 2013; Medawar-Aguilar et al., 2019; Salimi et al., 2023), stool (Zhang et al., 2022), and DNA samples (Alves et al., 2017; Gökce et al., 2016; Xu et al., 2013), supporting serological diagnosis as well as molecular detection of *T. gondii*. Echeverri et al. (2020) created a label-free electrochemical glycobiosensor detecting anti-GPI IgM and IgG antibodies in serum (LOD: 0.31 IU mL^−1^). Similarly, Takara et al. (2019) designed graphene oxide (GO)-polymer electrochemical sensors for serological detection of *T. gondii* specific IgG antibodies, whereas Medawar-Aguilar et al. (2019) introduced a microfluidic laser-induced fluorescence (LIF) immunosensor for the same purpose. Jiang et al. (2013) developed an Au-Fe₃O₄/graphene-based immunosensor for Tg-IgM, while Morovati et al. (2019) introduced a lateral flow biosensor targeting the dense granule antigen 7 (GRA7) for IgG detection (Table 2). In contrast to these assays, which detect host immunological responses. DNA-based biosensors suggest potential adaptability for detecting *T. gondii* directly in food matrices. Collectively, these methods show potential for detecting PPs in contaminated food and water, thereby strengthening public health surveillance of food- and waterborne transmission.

## 7 Limitations and future perspectives

### 7.1 Limitations

Despite promising advances in biosensor technologies for detecting foodborne pathogens, significant limitations constrain their practical applications and require urgent attention to realize their full potential.

#### 7.1.1 Electrochemical biosensors

While electrochemical sensors offer high sensitivity and low detection limits, their dependence on bulky electrochemical stations and need for warm-up period and polarization periods severely restrict their deployment for on-site and real-time applications (Dong et al., 2024). Moreover, bioreceptor stability remains a critical bottleneck due to the limited shelf life of antibodies and environmental instability; nucleic acid-based biosensors are constrained to DNA targets, and aptamers are sensitive to degradation by nucleases. Although integrating CRISPR/Cas with electrochemical DNA sensors has effectively improved both sensitivity and specificity (Wang et al., 2023), challenges persist in multiplex detection and seamless device integration, limiting their use in water and food matrices for multiple pathogen detection (Chen et al., 2022; Liu, 2025). Future research should focus on developing robust, multiplexed electrochemical platforms with stable bioreceptors and miniaturized, portable readout systems to enable their field applications.

#### 7.1.2 Optical biosensors

Optical systems, including fluorescence- and SPR-based sensors, provide higher sensitivity but are often bulky, expensive, and sensitive to environmental factors such as temperature variations and motion artifacts. Their extensive calibration requirements and susceptibility to spectral interference undermine reliability in dynamic food processing settings. Fluorescent biosensors, despite their sensitivity, require high-priced instrumentation, limiting accessibility in resource-constrained settings. Colorimetric biosensors are simple but highly influenced by environmental conditions, reducing their robustness (Dong et al., 2024). Research should prioritize the emergence of cost-effective, calibration-free optical biosensors with enhanced environmental tolerance and integration with smartphone-based systems, which facilitates POC application (Han et al., 2021; Kakkar et al., 2023).

#### 7.1.3 Piezoelectric sensors

Piezoelectric sensors show high thermal sensitivity and are better suited for dynamic monitoring; however, their application in detecting protozoa-specific indicators needs further exploration. This research gap highlights the need for targeted studies to adapt piezoelectric platforms for protozoan parasite detection, particularly when dealing with complex food samples (Feyziazar et al., 2022).

#### 7.1.4 Analytical versus operational sensitivity and sample preparation

Despite low LODs (as summarized in Table 1), most were derived from spiked samples tested in microliter-scale volumes (5–50 μL) using purified oocysts in PBS or deionized water, rather than naturally contaminated food or water samples. Only limited studies validated complex matrices: Nze et al. (2019) used hydrodynamic cavitation to process ground beef samples; Yu et al. (2021) tested patient stool samples; and Poitras et al. (2009) demonstrated 20–64% signal suppression from environmental contaminants, effects ignored in most LOD calculations. This gap reflects an unresolved sample-to-answer bottleneck. Furthermore, achieving reported sensitivities requires substantial pre-concentration, e.g., Luka et al. (2022) reported 20 oocysts/5 μL, necessitating concentration of ∼200 L of water to 5 μL to meet regulatory levels) via filtration, IMS, or centrifugation steps are rarely integrated into current biosensor platforms.

#### 7.1.5 Viability differentiation

Viability discrimination remains a critical limitation of conventional immunosensors and DNA-based sensors, which detect structural components persisting after cell death and thus cannot distinguish infectious from non-viable oocysts without additional processing (Brescia et al., 2009). However, emerging biosensor technologies are overcoming this constraint by targeting viability-specific biomarkers: EIS differentiates viable from heat-killed *C. parvum* via frequency-dependent impedance shifts reflecting membrane integrity (Dibao-Dina et al., 2015; Houssin et al., 2010); electrochemical detection of heat shock proteins (Hsp70) or nucleic acid sequence-based amplification (NASBA)-of viable-cell-specific rRNA enables metabolic activity assessment (Mahmudunnabi et al., 2024; Baeumner et al., 2001); and propidium monoazide (PMA) treatment combined with detection prevents signal generation from dead oocysts while preserving viable parasite signals (Brescia et al., 2009). Nevertheless, these biosensor-based viability correlates require rigorous calibration against gold-standard bioassays (mouse bioassay for *T. gondii*, cell culture for *Cryptosporidium*), as electrochemical signals may indicate membrane integrity without confirming infectivity, and most platforms remain at the proof-of-concept stage (Nze et al., 2019).

#### 7.1.6 General challenges in biosensor platforms

A pervasive issue is the limited validation of biosensors in complex food matrices, where matrix effects can compromise sensor sensitivity and specificity. This limitation underscores the importance of comprehensive testing under practical conditions and the development of strategies to eliminate these interferences (Quintela et al., 2022; Zhang et al., 2025). Additionally, integrating multi-sensing modalities into a single platform to enable parallel multiplex detection remains challenging due to the need to balance reaction kinetics, sensor size, and signal transduction efficiency (Kulkarni et al., 2023; Ullah et al., 2024).

The catalytic properties of nanomaterials such as gold‑palladium nanoparticles, copper(II)-reduced graphene oxide nanoparticles, AuNPs, iron metal-organic framework nanoparticles, silver nanocluster nanozymes, manganese dioxide nanoparticles, and manganese cobalt oxide offer promising avenues for straightforward colorimetric detection of diverse foodborne pathogens. However, operational stability, scalability, and cost effectiveness remain unresolved issues, particularly for large-scale adoption in resource-limited settings (Cambra-Pellejà et al., 2025; Kelly-Hope et al., 2018; Saldaña-Ahuactzi et al., 2025). The replacement of expensive gold electrodes with carbon-based materials enhances affordability, but requires long-term stability studies and the customer's training to ensure reliability (Cambra-Pellejà et al., 2025; Kelly-Hope et al., 2018).

Collectively, these limitations highlight the gap between proof-of-concept biosensor designs and their reliable deployment in food and water safety monitoring.

### 7.2 Future research directions and related gaps

To advance technology for foodborne pathogen detection, additional studies should be carried out to (i) enhance sensor stability and robustness against environmental and matrix interferences, (ii) develop multiplex platforms capable of detecting multiple pathogens simultaneously and differentiating live from dead pathogens, (iii) integrate biosensor outputs with advanced algorithms and artificial intelligence to improve biomedical applications and predictive capabilities (Gattani et al., 2023; Luka et al., 2022), (iv) focus on cost reduction and miniaturization to facilitate deployment in resource-constrained environments and ensure equitable access, (v) design mobile-based biosensors utilizing smartphones, cameras, and optical biosensors specific for the detection of foodborne pathogens, enabling real-time, on-site detection, (vi) address the standardization and regulatory gaps, especially for PPs. Further studies should prioritize testing the sensor with other potential interferants and examining the sensor's abilities by establishing ISO-compliant, validated biosensor protocols (Ahmadi et al., 2020; Haleem et al., 2021; Syedmoradi et al., 2017).

#### 7.2.1 Regulatory and funding challenges

The application of biosensor-based innovations into practical tools is further hindered by regulatory barriers and funding constraints (Bhandari et al., 2022; Casulli, 2021; Hotez et al., 2008). Increased awareness, interdisciplinary collaboration, and dedicated resource allocation are needed to scale up production and facilitate technology transfer. Critical challenges remain for widespread biosensor adoption in food safety surveillance, including a lack of standardized protocols for biosensor validation across diverse food and water matrices, insufficient data quality assurance frameworks, and limited interdisciplinary collaboration between sensor developers, food scientists, and regulatory bodies (Emma et al., 2024). Major gaps remain in tackling foodborne pathogen contamination due to the absence of standardized ISO methods for detecting pathogens such as *T. gondii* oocysts in fruits and vegetables. The WHO ASSURED criteria provide guidelines for developing efficient POC devices to identify major human diseases (Ahmadi et al., 2020; Syedmoradi et al., 2017), emphasizing affordability, sensitivity, specificity, and user-friendliness (Syedmoradi et al., 2017); however, research gaps exist regarding the detection of PPs in water and food matrices. Once regulatory, technical, and operational gaps are addressed, integration of biosensor-based detection into HACCP systems will follow as a logical step.

## 8 Conclusions

This review highlights biosensor-based technologies for the detection of zoonotic parasites, including *Cryptosporidium* spp. and *T. gondii* in complex matrices such as food and water, which may offer significant improvements in speed, precision, and portability. Emerging biosensors have remarkable potential for detecting minute concentrations of parasites, with some achieving a LOD as low as a single oocyst. Among these, electrochemical biosensors utilizing aptamers or antibodies as recognition elements have shown particularly promising results due to their higher sensitivity and affordability compared to spectroscopic and nucleic-based biosensors; however, their utilization at commercial levels has not yet been achieved. Innovative techniques such as CRISPR/Cas integration with smartphone-based readouts and the incorporation of nanomaterials further enhance sensitivity, enabling decentralized and real-time monitoring. Moreover, emerging electrochemical and optical biosensors detecting metabolic activity markers, heat shock proteins, or employing PMA-coupled detection, offer pathways to rapid viability screening, with NASBA-based methods achieving rapid detection. However, rigorous correlation of biosensor signals with infectivity assays is essential before these platforms can replace current gold standards in food safety surveillance. By bridging gaps in global food safety surveillance, biosensors hold great potential to mitigate the public health impact of foodborne parasites. Their integration into the HACCP system and POC diagnostics may revolutionize the food safety, ensuring safe food and water supplies and reducing the socioeconomic burden of cryptosporidiosis and toxoplasmosis.

## CRediT authorship contribution statement

**Munwar Ali:** Writing – review & editing, Writing – original draft, Visualization, Validation, Software, Investigation, Formal analysis, Data curation, Conceptualization. **Xiaohui Liang:** Writing – review & editing, Visualization, Data curation. **Ali Raza:** Writing – review & editing, Writing – original draft, Visualization, Data curation. **Chang Xu:** Writing – review & editing, Writing – original draft, Formal analysis. **Tingting Sun:** Writing – review & editing, Writing – original draft, Methodology. **Qing He:** Writing – review & editing, Formal analysis. **Zizye Zhu:** Writing – review & editing, Visualization, Investigation. **Kun Li:** Writing – review & editing, Writing – original draft, Supervision, Project administration, Funding acquisition, Formal analysis. **Lizeng Peng:** Writing – review & editing, Visualization, Resources, Project administration, Funding acquisition, Conceptualization.

## Funding

The study was partially supported by the 10.13039/501100001809National Natural Science Foundation of China (32102692), Innovation capability enhancement project of technology-based small and medium-sized enterprises of Shandong Province (2023TSGC0235), and Innovation capability enhancement project of technology-based small and medium-sized enterprises of Jinan City (Research on Key Technological Innovations for High-value Utilization of Rose rugosa cv. Plena through Probiotic Fermentation).

## Declaration of competing interest

The authors declare no conflict of interest.

## Data Availability

All the data is contained within the manuscript.

## References

[bb0005] Åberg R., Sjöman M., Hemminki K., Pirnes A., Räsänen S., Kalanti A. (2015). *Cryptosporidium parvum* caused a large outbreak linked to frisée salad in Finland, 2012. Zoonoses Public Health.

[bb0010] Abhari F.M., Niyyati M., Aghdaei H.A., Mirjalali H. (2023). Loop mediated isothermal amplification for detection of foodborne parasites: a journey from lab to lab-on-a-chip. Food Control.

[bb0015] Ahmadi A., Kabiri S., Omidfar K. (2020). Advances in HbA1c biosensor development based on field effect transistors: a review. IEEE Sensors J..

[bb0020] Al-Awadhi M., Iqbal J. (2025). Prevalence of parasitic contamination of locally grown and imported fresh leafy vegetables sold in an open market in Kuwait. Adv. Infect. Dis..

[bb0025] Algaba I.G., Geerts M., Jennes M., Coucke W., Opsteegh M., Cox E. (2017). A more sensitive, efficient and ISO 17025 validated magnetic capture real time PCR method for the detection of archetypal *Toxoplasma gondii* strains in meat. Int. J. Parasitol..

[bb0030] Al-Hindi R.R., Teklemariam A.D., Alharbi M.G., Alotibi I., Azhari S.A., Qadri I. (2022). Bacteriophage-based biosensors: a platform for detection of foodborne bacterial pathogens from food and environment. Biosensors.

[bb0035] Alves L.M., Rodovalho V.R., Castro A.C.H., Freitas M.A.R., Mota C.M., Mineo T.W. (2017). Development of direct assays for *Toxoplasma gondii* and its use in genomic DNA sample. J. Pharm. Biomed. Anal..

[bb0040] Amdouni Y., Hammami I., Farhat N., Rekik M., Gharbi M. (2024). First molecular and phylogenetic identification of *Toxoplasma gondii* in sheep liver intended for human consumption in northern Tunisia. Acta Parasitol..

[bb0045] Angus S.V., Kwon H.-J., Yoon J.-Y. (2012). Field-deployable and near-real-time optical microfluidic biosensors for single-oocyst-level detection of *Cryptosporidium parvum* from field water samples. J. Environ. Monit..

[bb0050] Angus S.V., Kwon H.-J., Yoon J.-Y. (2012). Optical Diagnostics and Sensing XII: Toward Point-of-Care Diagnostics; and Design and Performance Validation of Phantoms Used in Conjunction with Optical Measurement of Tissue IV.

[bb0055] Aramini J.J., Stephen C., Dubey J.P., Engelstoft C., Schwantje H., Ribble C.S. (1999). Potential contamination of drinking water with *Toxoplasma gondii* oocysts. Epidemiol. Infect..

[bb0060] Augendre L., Costa D., Escotte-Binet S., Aubert D., Villena I., Dumètre (2023). Surrogates of foodborne and waterborne protozoan parasites: a review. Food Waterborne Parasitol..

[bb0065] Baeumner A.J., Humiston M.C., Montagna R.A., Durst R.A. (2001). Detection of viable oocysts of *Cryptosporidium parvum* following nucleic acid sequence-based amplification. Anal. Chem..

[bb0070] Baldursson S., Karanis P. (2011). Waterborne transmission of protozoan parasites: review of worldwide outbreaks – an update 2004-2010. Water Res..

[bb0075] Banakar M., Hamidi M., Khurshid Z., Zafar M.S., Sapkota J., Azizian R. (2022). Electrochemical biosensors for pathogen detection: an updated review. Biosensors.

[bb0080] Batz M.B., Hoffmann S., Morris J.G. (2012). Ranking the disease burden of 14 pathogens in food sources in the United States using attribution data from outbreak investigations and expert elicitation. J. Food Prot..

[bb0085] Berrouch S., Escotte-Binet S., Amraouza Y., Flori P., Aubert D., Villena I. (2020). *Cryptosporidium* spp., *Giardia duodenalis* and *Toxoplasma gondii* detection in fresh vegetables consumed in Marrakech, Morocco. Afr. Health Sci..

[bb0090] Bhandari D., Chen F.-C., Bridgman R.C. (2022). Magnetic nanoparticles enhanced surface plasmon resonance biosensor for rapid detection of *Salmonella typhimurium* in romaine lettuce. Sensors.

[bb0095] Bourli P., Eslahi A.V., Tzoraki O., Karanis P. (2023). Waterborne transmission of protozoan parasites: a review of worldwide outbreaks – an update 2017-2022. J. Water Health.

[bb0100] Bouwknegt M., Devleesschauwer B., Graham H., Robertson L.J., van der Giessen J.W.V., Particiapants E.W. (2018). Prioritisation of food-borne parasites in Europe, 2016. Eurosurveillance.

[bb0105] Bowie W.R., King A.S., Werker D.H., Isaac-Renton J.L., Bell A., Eng S.B. (1997). Outbreak of toxoplasmosis associated with municipal drinking water. Lancet.

[bb0110] Brescia C.C., Griffin S.M., Ware M.W., Varughese E.A., Egorov A.I., Villegas E.N. (2009). *Cryptosporidium* propidium monoazide-PCR, a molecular biology-based technique for genotyping of viable *Cryptosporidium* oocysts. Appl. Environ. Microbiol..

[bb0115] Byrne B., Stack E., Gilmartin N., O’Kennedy R. (2009). Antibody-based sensors: principles, problems and potential for detection of pathogens and associated toxins. Sensors.

[bb0120] Cali K., Tuccori E., Persaud K.C. (2020). Gravimetric biosensors. Methods Enzymol..

[bb0125] Cambra-Pellejà M., van Lieshout L., Baptista-Pires L., Vilaplana M., Muñoz J., Gandasegui J. (2025). Crucial role of biosensors in the detection of helminth biomarkers in public health programmes. Lancet Microbe.

[bb0130] Campbell G.A., Mutharasan R. (2008). Near real-time detection of *Cryptosporidium parvum* oocyst by IgM-functionalized piezoelectric-excited millimeter-sized cantilever biosensor. Biosens. Bioelectron..

[bb0135] Carstens C.K., Salazar J.K., Darkoh C. (2019). Multistate outbreaks of foodborne illness in the United States associated with fresh produce from 2010 to 2017. Front. Microbiol..

[bb0140] Casulli A. (2021). New global targets for NTDs in the WHO roadmap 2021–2030. PLoS Negl. Trop. Dis..

[bb0145] CDC (Centers for Disease Control and Prevention) (2019). https://www.cdc.gov/healthyswimming/data-research/cryptosporidiosis-surveillance.html.

[bb0150] Chalmers R.M., Robertson L.J., Dorny P., Jordan S., Kärssin A., Katzer F. (2020). Parasite detection in food: current status and future needs for validation. Trends Food Sci. Technol..

[bb0155] Chang Y., Wang Y., Zhang J., Xing Y., Li G., Deng D. (2022). Overview on the design of magnetically assisted electrochemical biosensors. Biosensors.

[bb0160] Chen C., Wang J. (2020). Optical biosensors: an exhaustive and comprehensive review. Analyst.

[bb0165] Chen D., Lin J., Liu Y., Li X., Chen G., Hua Q. (2016). Identification of TgAtg8–TgAtg3 interaction in *Toxoplasma gondii*. Acta Trop..

[bb0170] Chen K., Shen Z., Wang G., Gu W., Zhao S., Lin Z. (2022). Research progress of CRISPR-based biosensors and bioassays for molecular diagnosis. Front. Bioeng. Biotechnol..

[bb0175] Chen H., Luo B., Wu S., Shi S., Dai Q., Peng Z. (2023). Microfluidic biosensor based on molybdenum disulfide (MoS2) modified thin-core microfiber for immune detection of *Toxoplasma gondii*. Sensors.

[bb0180] Connelly J.T., Nugen S.R., Borejsza-Wysocki W., Durst R.A., Montagna R.A., Baeumner A.J. (2008). Human pathogenic *Cryptosporidium* species bioanalytical detection method with single oocyst detection capability. Anal. Bioanal. Chem..

[bb0185] Corso P.S., Kramer M.H., Blair K.A., Addiss D.G., Davis J.P., Haddix A.C. (2003). Cost of illness in the 1993 waterborne *Cryptosporidium* outbreak, Milwaukee, Wisconsin. Emerg. Infect. Dis..

[bb0190] Crivianu-Gaita V., Thompson M. (2016). Aptamers, antibody scFv, and antibody Fab’fragments: an overview and comparison of three of the most versatile biosensor biorecognition elements. Biosens. Bioelectron..

[bb0195] Daher M.G., Taya S.A., Faragallah O.S., Patel S.K., Prajapati Y.K., Armghan A. (2025). Detection of protozoa in drinking water using SPR biosensor employing titanium dioxide and MXene nanomaterial. J. Comput. Electron..

[bb0200] Daramola O.B., Omole R.K., Akinsanola B.A. (2025). Emerging applications of biorecognition elements-based optical biosensors for food safety monitoring. Discov. Sens..

[bb0205] Davis F., Higson S.P.J. (2012). Biosensors for Medical Applications (Woodhead Publishing Series in Biomaterials).

[bb0210] de Moura L., Bahia-Oliveira L.M.G., Marcelo Y., Wada J.L.J., Suely H., Tuboi E.H.C. (2006). Waterborne toxoplasmosis, Brazil, from field to gene. Emerg. Infect. Dis..

[bb0215] Debierre-Grockiego F., Niehus S., Coddeville B., Elass E., Poirier F., Weingart R. (2010). Binding of *Toxoplasma gondii* glycosylphosphatidylinositols to galectin-3 is required for their recognition by macrophages. J. Biol. Chem..

[bb0220] Di Nardo F., Anfossi L. (2020). Commercial Biosensors and their Applications.

[bb0225] Dibao-Dina A., Follet J., Ibrahim M., Vlandas A., Senez V. (2015). Electrical impedance sensor for quantitative monitoring of infection processes on HCT-8 cells by the waterborne parasite *Cryptosporidium*. Biosens. Bioelectron..

[bb0230] Dong X., Huang A., He L., Cai C., You T. (2024). Recent advances in foodborne pathogen detection using photoelectrochemical biosensors: from photoactive material to sensing strategy. Front. Sustain. Food Syst..

[bb0235] Dubey J.P. (2004). Toxoplasmosis – a waterborne zoonosis. Vet. Parasitol..

[bb0240] Dubey J.P. (2021).

[bb0245] Dumètre A., Dubey J.P., Ferguson D.J.P., Bongrand P., Azas N., Puech P.H. (2013). Mechanics of the *Toxoplasma gondii* oocyst wall. Proc. Natl. Acad. Sci. USA.

[bb0250] Echeverri D., Garg M., Silva D.V., Orozco J. (2020). Phosphoglycan-sensitized platform for specific detection of anti-glycan IgG and IgM antibodies in serum. Talanta.

[bb0255] Efstratiou A., Ongerth J., Karanis P. (2017). Evolution of monitoring for *Giardia* and *Cryptosporidium* in water. Water Res..

[bb0260] Emma B., Chan K., Bridle H., England P., Gaura E., Halford A. (2024).

[bb0265] Eslahi A.V., Mamedova S., Nassiba R., Karanis P. (2024). Unveiling risks in healthy food: vegetables and fruits are linked to the distribution chain of protozoan parasites. Food Microbiol..

[bb0270] Evans A.E.V., Mateo-Sagasta J., Qadir M., Boelee E., Ippolito A. (2019). Agricultural water pollution: key knowledge gaps and research needs. Curr. Opin. Environ. Sustain..

[bb0275] Eyvazi S., Baradaran B., Mokhtarzadeh A., de la Guardia M. (2021). Recent advances on development of portable biosensors for monitoring of biological contaminants in foods. Trends Food Sci. Technol..

[bb0280] FAO (2021). Food Safety Technical Toolkit for Asia and the Pacific.

[bb0285] Fayer R., Morgan U., Upton S.J. (2000). Epidemiology of *Cryptosporidium*: transmission, detection and identification. Int. J. Parasitol..

[bb0290] Felix F.S., Angnes L. (2018). Electrochemical immunosensors – a powerful tool for analytical applications. Biosens. Bioelectron..

[bb0295] Ferreira F.P., Caldart E.T., Freire R.L., Mitsuka-Breganó R., de Freitas F.M., Miura A.C., Mareze M. (2018). The effect of water source and soil supplementation on parasite contamination in organic vegetable gardens. Rev. Bras. Parasitol. Vet..

[bb0300] Feyziazar M., Amini M., Jahanban-Esfahlan A., Baradaran B., Oroojalian F., Kamrani A. (2022). Recent advances on the piezoelectric, electrochemical, and optical biosensors for the detection of protozoan pathogens. TrAC Trends Anal. Chem..

[bb0305] Fradette M.S., Culley A.I., Charette S.J. (2022). Detection of *Cryptosporidium* spp. and *Giardia* spp. in environmental water samples: a journey into the past and new perspectives. Microorganisms.

[bb0310] Gabriël S., Dorny P., Saelens G., Dermauw V. (2022). Foodborne parasites and their complex life cycles challenging food safety in different food chains. Foods.

[bb0315] Gaft M., Reisfeld R., Panczer G. (2015). https://link.springer.com/book/10.1007/978-3-319-24765-6.

[bb0320] Garcia L.S., Shimizu R.Y. (1997). Evaluation of nine immunoassay kits (enzyme immunoassay and direct fluorescence) for detection of *Giardia lamblia* and *Cryptosporidium parvum* in human fecal specimens. J. Clin. Microbiol..

[bb0325] Gattani A., Mandal S., Khan M.H., Jain A., Ceaser D., Mishra A. (2023). Novel electrochemical biosensing for detection of neglected tropical parasites of animal origin: recent advances. Electroanalysis.

[bb0330] Gaulin C., Ramsay D., Thivierge K., Tataryn J., Courville A., Martin C., Cunningham P. (2020). Acute toxoplasmosis among Canadian deer hunters associated with consumption of undercooked deer meat hunted in the United States. Emerg. Infect. Dis..

[bb0335] Gharpure R., Perez A., Miller A.D., Wikswo M.E., Silver R., Hlavsa M.C. (2019). Cryptosporidiosis outbreaks — United States, 2009–2017. MMWR Morb. Mortal Wkly. Rep..

[bb0340] Gökce G., Erdem A., Ceylan C., Akgöz M. (2016). Voltammetric detection of sequence-selective DNA hybridization related to *Toxoplasma gondii* in PCR amplicons. Talanta.

[bb0345] González-Ramírez L.C., Djabayan-Djibeyan P., Prato J.G., Ríos C.A.G., Carrero J.C., Trelis M. (2024). Field study of parasitic contamination of fruits, vegetables and leafy greens in the Ecuadorian Andes. F1000Res.

[bb0350] Graczyk T.K., Cranfield M.R., Fayer R. (1996). Evaluation of commercial enzyme immunoassay (EIA) and immunofluorescent antibody (FA) test kits for detection of *Cryptosporidium* oocysts of species other than *Cryptosporidium parvum*. Am. J. Trop. Med. Hyg..

[bb0355] Gu X., Lei L., Sun Y., Si X., Wang M., Li F. (2019). Microfluidic diffraction phase microscopy for high-throughput, artifact-free quantitative phase imaging and identification of waterborne parasites. Opt. Laser Technol..

[bb0360] Hai X., Li Y., Zhu C., Song W., Cao J., Bi S. (2020). DNA-based label-free electrochemical biosensors: from principles to applications. TrAC Trends Anal. Chem..

[bb0365] Haleem A., Javaid M., Singh R.P., Suman R., Rab S. (2021). Biosensors applications in medical field: a brief review. Sens. Int..

[bb0370] Han X., Liu Y., Yin J., Yue M., Mu Y. (2021). Microfluidic devices for multiplexed detection of foodborne pathogens. Food Res. Int..

[bb0375] Hasanzadeh M., Sahmani R., Solhi E., Mokhtarzadeh A., Shadjou N., Mahboob S. (2018). Ultrasensitive immunoassay of carcinoma antigen 125 in untreated human plasma samples using gold nanoparticles with flower like morphology: a new platform in early stage diagnosis of ovarian cancer and efficient management. Int. J. Biol. Macromol..

[bb0380] Hassan E.M., Dixon B.R., Sattar S.A., Stalker A., Örmeci B., DeRosa M.C. (2021). Highly sensitive magnetic-microparticle-based aptasensor for *Cryptosporidium parvum* oocyst detection in river water and wastewater: effect of truncation on aptamer affinity. Talanta.

[bb0385] Hassan E.M., Örmeci B., DeRosa M.C., Dixon B.R., Sattar S.A., Iqbal A. (2021). A review of *Cryptosporidium* spp. and their detection in water. Water Sci. Technol..

[bb0390] Havelaar A.H., Haagsma J.A., Mangen M.J.J., Kemmeren J.M., Verhoef L.P.B., Vijgen S.S.M.C. (2012). Disease burden of foodborne pathogens in the Netherlands 2009. Int. J. Food Microbiol..

[bb0395] He L., Ni L., Zhang X., Zhang C., Li R., Xu S. (2015). Fluorescent detection of specific DNA sequences related to *Toxoplasma gondii* based on magnetic fluorescent nanoparticles Fe3O4/CdTe biosensor. Int. J. Biochem. Res. Rev..

[bb0400] Hianik T., Spagnolo S., Thompson M. (2024). Trends in development of aptamer-based biosensor technology for detection of bacteria. Adv. Biochem. Eng. Biotechnol..

[bb0405] Hjort R.G., Pola C.C., Soares R.R.A., Oliveira D.A., Stromberg L., Claussen J.C. (2024). Encyclopedia of Food Safety.

[bb0410] Hoffmann S., Batz M.B., Morris J.G. (2012). Annual cost of illness and quality-adjusted life year losses in the United States due to 14 foodborne pathogens. J. Food Prot..

[bb0415] Hosu O., Selvolini G., Marrazza G. (2018). Recent advances of immunosensors for detecting food allergens. Curr. Opin. Electrochem..

[bb0420] Hotez P.J., Brindley P.J., Bethony J.M., King C.H., Pearce E.J., Jacobson J. (2008). Helminth infections: the great neglected tropical diseases. J. Clin. Invest..

[bb0425] Houssin T., Follet J., Follet A., Dei-Cas E., Senez V. (2010). Label-free analysis of water-polluting parasite by electrochemical impedance spectroscopy. Biosens. Bioelectron..

[bb0430] Huang W., Yang L., Lei L., Li F. (2017). Label-free detection and identification of waterborne parasites using a microfluidic multi-angle laser scattering system. Opt. Commun..

[bb0435] Huang L., Zhang C., Ye R., Yan B., Zhou X., Xu W. (2024). Capacitive biosensors for label-free and ultrasensitive detection of biomarkers. Talanta.

[bb0440] Hussain M.A., Stitt V., Szabo E.A., Nelan B. (2017). *Toxoplasma gondii* in the food supply. Pathogens.

[bb0445] Ilkhani H., Zhang H., Zhou A. (2019). A novel three-dimensional microTAS chip for ultra-selective single base mismatched *Cryptosporidium* DNA biosensor. Sens. Actuators B.

[bb0450] Iqbal A., Labib M., Muharemagic D., Sattar S., Dixon B.R., Berezovski M.V. (2015). Detection of *Cryptosporidium parvum* oocysts on fresh produce using DNA aptamers. PLoS One.

[bb0455] Iqbal A., Liu J., Dixon B., Zargar B., Sattar S.A. (2019). Development and application of DNA-aptamer-coupled magnetic beads and aptasensors for the detection of *Cryptosporidium parvum* oocysts in drinking and recreational water resources. Can. J. Microbiol..

[bb0460] ISO (International Organization for Standardization) (2016). https://www.iso.org/standard/54870.html.

[bb0465] ISO (International Organization for Standardization) (2016). https://www.iso.org/standard/63252.html.

[bb0470] IUPAC (2006). Compendium of Chemical Terminology, 2nd Ed. (the "Gold Book"). Compiled by A.D. McNaught and A. Wilkinson. Blackwell Scientific Publications, Oxford. XML On-Line Corrected Version (2006–2019) Created by M. Nic, J. Jirat and B. Kosata; Updates Compiled by A. Jenkins. Interactive Version (2019–) Created by S.J. Chalk; Content Edited by J. Kaiser. https://goldbook.iupac.org/.

[bb0475] Jiang S., Hua E., Liang M., Liu B., Xie G. (2013). A novel immunosensor for detecting *Toxoplasma gondii*-specific IgM based on goldmag nanoparticles and graphene sheets. Colloids Surf. B: Biointerfaces.

[bb0480] Kakkar S., Gupta P., Kumar N., Kant K. (2023). Progress in fluorescence biosensing and food safety towards point-of-detection (pod) system. Biosensors.

[bb0485] Kang C.D., Lee S.W., Park T.H., Sim S.J. (2006). Performance enhancement of real-time detection of protozoan parasite, *Cryptosporidium* oocyst by a modified surface plasmon resonance (SPR) biosensor. Enzym. Microb. Technol..

[bb0490] Kang C.D., Cao C., Lee J., Choi I.S., Kim B.W., Sim S.J. (2008). Surface plasmon resonance-based inhibition assay for real-time detection of *Cryptosporidium parvum* oocyst. Water Res..

[bb0495] Karanis P., Kourenti C., Smith H. (2007). Waterborne transmission of protozoan parasites: a worldwide review of outbreaks and lessons learnt. J. Water Health.

[bb0500] Kelly-Hope L.A., Blundell H.J., Macfarlane C.L., Molyneux D.H. (2018). Innovative surveillance strategies to support the elimination of filariasis in Africa. Trends Parasitol..

[bb0505] Khalil I.A., Troeger C., Rao P.C., Blacker B.F., Brown A., Brewer T.G. (2018). Morbidity, mortality, and long-term consequences associated with diarrhoea from *Cryptosporidium* infection in children younger than 5 years: a meta-analyses study. Lancet Glob. Health.

[bb0510] Khare R., Verma S., Singh P., Pal S., Shrivastava R. (2022). Blueprint for impedance-based electrochemical biosensors as bioengineered tools in the field of nano-diagnostics. Curr. Res. Biotechnol..

[bb0515] Kotloff K.L., Nataro J.P., Blackwelder W.C., Nasrin D., Farag T.H., Panchalingam S. (2013). Burden and aetiology of diarrhoeal disease in infants and young children in developing countries (the global enteric multicenter study, GEMS): a prospective, case-control study. Lancet.

[bb0520] Kramer M.F., Vesey G., Look N.L., Herbert B.R., Simpson-Stroot J.M., Lim D.V. (2007). Development of a *Cryptosporidium* oocyst assay using an automated fiber optic-based biosensor. J. Biol. Eng..

[bb0525] Kulkarni M.B., Ayachit N.H., Aminabhavi T.M. (2023). Recent advances in microfluidics-based electrochemical sensors for foodborne pathogen detection. Biosensors.

[bb0530] Laczka O., Skillman L., Ditcham W.G., Hamdorf B., Wong D.K.Y., Bergquist P. (2013). Application of an ELISA-type screen printed electrode-based potentiometric assay to the detection of *Cryptosporidium parvum* oocysts. J. Microbiol. Methods.

[bb0535] Larsen T.G., Etheberg S., Nielson H.L., Hartmeyer G.N., Nielsen L., Zangenberg M. (2025). From rare to recognized: enhanced detection uncovers *Cryptosporidium* endemicity and species diversity in Denmark. Emerg. Microbes Infect..

[bb0540] Lass A., Ma L., Kontogeorgos I., Zhang X., Li X., Karanis P. (2019). First molecular detection of *Toxoplasma gondii* in vegetable samples in China using qualitative, quantitative real-time PCR and multilocus genotyping. Sci. Rep..

[bb0545] Lei R., Li L., Wu P., Fei X., Zhang Y., Wang J. (2022). RPA/CRISPR/Cas12a-based on-site and rapid nucleic acid detection of *Toxoplasma gondii* in the environment. ACS Synth. Biol..

[bb0550] Li J., Wang Z., Karim M.R., Zhang L. (2020). Detection of human intestinal protozoan parasites in vegetables and fruits: a review. Parasit. Vectors.

[bb0555] Li X., Zhang X., Jian Y., Wang G., Ma L. (2020). Detection of *Cryptosporidium* oocysts and *Giardia* cysts in vegetables from street markets from the Qinghai Tibetan plateau area in China. Parasitol. Res..

[bb0560] Li Y., Deng F., Hall T., Vesey G., Goldys E.M. (2021). CRISPR/Cas12a-powered immunosensor suitable for ultra-sensitive whole *Cryptosporidium* oocyst detection from water samples using a plate reader. Water Res..

[bb0565] Liang C., Xu S., Yang J., Zhang J., Dai Z., Sun B. (2009). A biosensing of *Toxoplasma gondii* DNA with CdTe/Fe 3 O 4 dual functional quantum dot as reporter group. Second Int. Conf. Smart Mater. Nanotechnol. Eng..

[bb0570] Liao Z., Zhang Y., Li Y., Miao Y., Gao S., Lin F. (2019). Microfluidic chip coupled with optical biosensors for simultaneous detection of multiple analytes: a review. Biosens. Bioelectron..

[bb0575] Liu G. (2025). Advancing CRISPR/Cas biosensing with integrated devices. ACS Sens..

[bb0580] Liu Q., Wang Z.-D., Huang S.-Y., Zhu X.-Q. (2015). Diagnosis of toxoplasmosis and typing of *Toxoplasma gondii*. Parasit. Vectors.

[bb0585] Liu Q., Zhang W., Chen S., Zhuang Z., Zhang Y., Jiang L. (2020). SELEX tool: a novel and convenient gel-based diffusion method for monitoring of aptamer-target binding. J. Biol. Eng..

[bb0590] Luka G., Ahmadi A., Najjaran H., Alocilja E., DeRosa M., Wolthers K. (2015). Microfluidics integrated biosensors: a leading technology towards lab-on-a-chip and sensing applications. Sensors.

[bb0595] Luka G., Samiei E., Dehghani S., Johnson T., Najjaran H. (2019). Label-free capacitive biosensor for detection of *Cryptosporidium*. Sensors.

[bb0600] Luka G.S., Nowak E., Toyata Q.R., Tasnim N., Najjaran H., Hoorfar M. (2021). Portable on-chip colorimetric biosensing platform integrated with a smartphone for label/PCR-free detection of *Cryptosporidium* RNA. Sci. Rep..

[bb0605] Luka G.S., Najjaran H., Hoorfar M. (2022). On-chip-based electrochemical biosensor for the sensitive and label-free detection of *Cryptosporidium*. Sci. Rep..

[bb0610] Mac Kenzie W.R., Hoxie N.J., Proctor M.E., Gradus M.S., Blair K.A., Peterson D.E., Kazmierczak J.J., Addiss D.G., Fox K.R., Rose J.B. (1994). A massive outbreak in Milwaukee of *Cryptosporidium* infection transmitted through the public water supply. N. Engl. J. Med..

[bb0615] Mahmudunnabi R.G., Kasetsirikul S., Soda N., Sallam M., Pannu A.S., Nguyen N.T. (2024). Critical evaluation of current isolation, detection, and genotyping methods of *Cryptosporidium* species and future direction. Environ. Sci. Water Res. Technol..

[bb0620] Mahmudunnabi R.G., Pannu A.S., Nguyen N.-T., Stratton H.M., Shiddiky M.J.A. (2025). Avoiding commercial kit-based DNA isolation and purification steps: a rapid method for *Cryptosporidium* oocyst detection. Sens. Diagn..

[bb0625] Marín-García P.-J., Planas N., Llobat L. (2022). *Toxoplasma gondii* in foods: prevalence, control, and safety. Foods.

[bb0630] Marques C.S., Sousa S., Castro A., da Costa J.M.C. (2020). Detection of *Toxoplasma gondii* oocysts in fresh vegetables and berry fruits. Parasit. Vectors.

[bb0635] Martinez M.P., Carmena D., Herrador B.R.G., Miguel M.P., Campelli G.S., Álvarez R.M.G. (2024). Marked increase in cryptosporidiosis cases, Spain, 2023. Eurosurveillance.

[bb0640] Mateo-Sagasta J., Zadeh S.M., Turral H., Burke J. (2017).

[bb0645] Medawar-Aguilar V., Jofre C.F., Fernández-Baldo M.A., Alonso A., Angel S., Raba J. (2019). Serological diagnosis of toxoplasmosis disease using a fluorescent immunosensor with chitosan-ZnO-nanoparticles. Anal. Biochem..

[bb0650] Mi F., Guan M., Hu C., Peng F., Sun S., Wang X. (2021). Application of lectin-based biosensor technology in the detection of foodborne pathogenic bacteria: a review. Analyst.

[bb0655] Monge S., Pijnacker R., Van Pelt W., Franz E. (2019). Accounting for long-term manifestations of *Cryptosporidium* spp infection in burden of disease and cost-of-illness estimations, the Netherlands (2013–2017). PLoS One.

[bb0660] Montoya J.G., Liesenfeld O. (2004). Toxoplasmosis. Lancet.

[bb0665] Moreno-Mesonero L., Soler L., Amorós I., Moreno Y., Ferrús M.A., Alonso J.L. (2023). Protozoan parasites and free-living amoebae contamination in organic leafy green vegetables and strawberries from Spain. Food Waterborne Parasitol..

[bb0670] Morovati H., Seyyed Tabaei S.J., Gholamzad M., Omidfar K., Ahmadi A., Arab Mazar Z. (2019). Development of a lateral flow immunoassay using recombinant dense granular antigen (GRA) 7 to detect anti-*Toxoplasma gondii* IgG antibodies. Arch. Razi Inst..

[bb0675] Naguleswaran A., Fialho A.M., Chaudhari A., Hong C.S., Chakrabarty A.M., Sullivan W.J. (2008). Azurin-like protein blocks invasion of *Toxoplasma gondii* through potential interactions with parasite surface antigen SAG1. Antimicrob. Agents Chemother..

[bb0680] Nemati S., Shalileh F., Mirjalali H., Omidfar K. (2023). Toward waterborne protozoa detection using sensing technologies. Front. Microbiol..

[bb0685] Ng-Hublin J.S.Y., Combs B., Reid S., Ryan U. (2018). Comparison of three cryptosporidiosis outbreaks in Western Australia: 2003, 2007 and 2011. Epidemiol. Infect..

[bb0690] Nugen S.R., Asiello P.J., Connelly J.T., Baeumner A.J. (2009). PMMA biosensor for nucleic acids with integrated mixer and electrochemical detection. Biosens. Bioelectron..

[bb0695] Nze U.C., Beeman M.G., Lambert C.J., Salih G., Gale B.K., Sant H.J. (2019). Hydrodynamic cavitation for the rapid separation and electrochemical detection of *Cryptosporidium parvum* and *Escherichia coli* O157: H7 in ground beef. Biosens. Bioelectron..

[bb0700] Olaru A., Bala C., Jaffrezic-Renault N., Aboul-Enein H.Y. (2015). Surface plasmon resonance (SPR) biosensors in pharmaceutical analysis. Crit. Rev. Anal. Chem..

[bb0705] O’Leary J.K., Sleator R.D., Lucey B. (2021). *Cryptosporidium* spp. diagnosis and research in the 21st century. Food Waterborne Parasitol..

[bb0710] Opsteegh M., Dam-Deisz C., de Boer P., DeCraeye S., Faré A., Hengeveld P. (2020). Methods to assess the effect of meat processing on viability of *Toxoplasma gondii*: towards replacement of mouse bioassay by *in vitro* testing. Int. J. Parasitol..

[bb0715] Park C.K., Kang C.D., Sim S.J. (2008). Non-labeled detection of waterborne pathogen *Cryptosporidium parvum* using a polydiacetylene-based fluorescence chip. Biotechnol. J. Healthc. Nutr. Technol..

[bb0720] Poitras C., Fatisson J., Tufenkji N. (2009). Real-time microgravimetric quantification of *Cryptosporidium parvum* in the presence of potential interferents. Water Res..

[bb0725] Quintela I.A., Vasse T., Lin C.-S., Wu V.C.H. (2022). Advances, applications, and limitations of portable and rapid detection technologies for routinely encountered foodborne pathogens. Front. Microbiol..

[bb0730] Ramanathan K., Danielsson B. (2001). Principles and applications of thermal biosensors. Biosens. Bioelectron..

[bb0735] Reiling S.J., Merks H., Zhu S., Boone R., Corneau N., Dixon B.R. (2021). A cloth-based hybridization array system for rapid detection of the food- and waterborne protozoan parasites *Giardia duodenalis*, *Cryptosporidium* spp. and *Toxoplasma gondii*. Food Waterborne Parasitol..

[bb0740] Reinholt S.J., Behrent A., Greene C., Kalfe A., Baeumner A.J. (2014). Isolation and amplification of mrna within a simple microfluidic lab on a chip. Anal. Chem..

[bb0745] Reischl U., Bretagne S., Krüger D., Ernault P., Costa J.M. (2003). Comparison of two DNA targets for the diagnosis of toxoplasmosis by real-time PCR using fluorescence resonance energy transfer hybridization probes. BMC Infect. Dis..

[bb0750] Ruiz-andino I.R. (2022). Examination of Binding Elements and Conditions of *Cryptosporidium parvum* Oocysts to Assess its Detection Potential in Water. https://dspacemainprd01.lib.uwaterloo.ca/server/api/core/bitstreams/6b16d4fa-b50c-4066-93de-b433f0166f10/content.

[bb0755] Rule K.L., Vikesland P.J. (2009). Surface-enhanced resonance Raman spectroscopy for the rapid detection of *Cryptosporidium parvum* and *Giardia lamblia*. Environ. Sci. Technol..

[bb0760] Safarpour H., Dehghani S., Nosrati R., Zebardast N., Alibolandi M., Mokhtarzadeh A. (2020). Optical and electrochemical-based nano-aptasensing approaches for the detection of circulating tumor cells (CTCs). Biosens. Bioelectron..

[bb0765] Saldaña-Ahuactzi Z., Gómez-Montaño F.J., Morales-Chávez J., Salinas R.A., Reyes-Betanzo C., Rojas-López M. (2025). Advancing foodborne pathogen detection: a review of traditional and innovative optical and electrochemical biosensing approaches. Microchim. Acta.

[bb0770] Salimi M., Keshavarz-Valian H., Mohebali M., Geravand M., Adabi M., Shojaee S. (2023). Electrochemical immunosensor based on carbon nanofibers and gold nanoparticles for detecting anti-*Toxoplasma gondii* IgG antibodies. Microchim. Acta.

[bb0775] Sande M.G., Rodrigues J.L., Ferreira D., Silva C.J., Rodrigues L.R. (2021). Novel biorecognition elements against pathogens in the design of state-of-the-art diagnostics. Biosensors.

[bb0780] Santos A., Carvalho F.C., Roque-Barreira M.-C., Zorzetto-Fernandes A.L., Gimenez-Romero D., Monzó I. (2015). Evidence for conformational mechanism on the binding of TgMIC4 with β-galactose-containing carbohydrate ligand. Langmuir.

[bb0785] Sarkar R., Tate J.E., Ajjampur S.S.R., Kattula D., John J., Ward H.D. (2014).

[bb0790] Sarkhosh T., Mayerberger E., Jellison K., Jedlicka S. (2021). Development of cell-imprinted polymer surfaces for *Cryptosporidium* capture and detection. Water Res..

[bb0795] Schoeps A., Röbl K., Walter N., Neute A., Walter B., Freudenau I. (2024). Increased number of cryptosporidiosis cases with travel history to Croatia might be related to swimming pools, Germany, 2023. Eurosurveillance.

[bb0800] Schumacher A.C., Elbadawi L.I., DeSalvo T., Straily A., Ajzenberg D., Letzer D. (2021). Toxoplasmosis outbreak associated with *Toxoplasma gondii*-contaminated venison—high attack rate, unusual clinical presentation, and atypical genotype. Clin. Infect. Dis..

[bb0805] Siavash Moakhar R., Mahimkar R., Khorrami Jahromi A., Mahshid S.S., del Real Mata C., Lu Y. (2023). Aptamer-based electrochemical microfluidic biosensor for the detection of *Cryptosporidium parvum*. ACS Sens..

[bb0810] Singh A., Sharma A., Ahmed A., Sundramoorthy A.K., Furukawa H., Arya S. (2021). Recent advances in electrochemical biosensors: applications, challenges, and future scope. Biosensors.

[bb0815] Siwak A.M., Baker P.G., Dube A. (2023). Biosensors as early warning detection systems for waterborne *Cryptosporidium*. Water Sci. Technol..

[bb0820] Sow S.O., Muhsen K., Nasrin D., Blackwelder W.C., Wu Y., Farag T.H. (2016). The burden of *Cryptosporidium* diarrheal disease among children < 24 months of age in moderate/high mortality regions of sub-Saharan Africa and South Asia, utilizing data from the global enteric multicenter study (GEMS). PLoS Negl. Trop. Dis..

[bb0825] Subauste C.S. (2019). Interplay between *Toxoplasma gondii*, autophagy, and autophagy proteins. Front. Cell. Infect. Microbiol..

[bb0830] Suleiman A.J., Mavrides D.E., Maxamhud S., Gentekaki E., Tsaousis A.D. (2024). Presence of *Cryptosporidium parvum* in pre-washed vegetables from different supermarkets in South East England: a pilot study. Parasitol. Res..

[bb0835] Syed Z. ul Q., Samaraweera S., Wang Z., Schrader J.K., Scott C., Schut J. (2024). Colorimetric hybridization sensor for DNA mimic of a SARS-CoV-2 RNA marker: direct and inverse bioanalysis. ACS Meas. Sci. Au.

[bb0840] Syedmoradi L., Daneshpour M., Alvandipour M., Gomez F.A., Hajghassem H., Omidfar K. (2017). Point of care testing: the impact of nanotechnology. Biosens. Bioelectron..

[bb0845] Taguchi T., Arakaki A., Takeyama H., Haraguchi S., Yoshino M., Kaneko M. (2007). Detection of *Cryptosporidium parvum* oocysts using a microfluidic device equipped with the SUS micromesh and FITC-labeled antibody. Biotechnol. Bioeng..

[bb0850] Takara E.A., Pereira S.V., Scala-Benuzzi M.L., Fernández-Baldo M.A., Raba J., Messina G.A. (2019). Novel electrochemical sensing platform based on a nanocomposite of PVA/PVP/RGO applied to IgG anti-*Toxoplasma gondii* antibodies quantitation. Talanta.

[bb0855] Techakasikornpanich M., Jangpatarapongsa K., Polpanich D., Zine N., Errachid A., Elaissari A. (2024). Biosensor technologies: DNA-based approaches for foodborne pathogen detection. TrAC Trends Anal. Chem..

[bb0860] Thiruppathiraja C., Kamatchiammal S., Adaikkappan P., Alagar M. (2011). An advanced dual labeled gold nanoparticles probe to detect *Cryptosporidium parvum* using rapid immuno-dot blot assay. Biosens. Bioelectron..

[bb0865] Thiruppathiraja C., Saroja V., Kamatchiammal S., Adaikkappan P., Alagar M. (2011). Development of electrochemical based sandwich enzyme linked immunosensor for *Cryptosporidium parvum* detection in drinking water. J. Environ. Monit..

[bb0870] Thompson A., Hable B., Young K., Hansen T., Strickler J.R., Silva M.R. (2020). Optically based hand-held sensor for visualization and quantification of *Cryptosporidium parvum*. Sens. Imaging.

[bb0875] Torgerson P.R., Devleesschauwer B., Praet N., Speybroeck N., Willingham A.L., Kasuga F., Rokni M.B. (2015). World Health Organization estimates of the global and regional disease burden of 11 foodborne parasitic diseases, 2010: a data synthesis. PLoS Med..

[bb0880] Trelis M., Sáez-Durán S., Puchades P., Castro N., Miquel A., Gozalbo M. (2022). Survey of the occurrence of *Giardia duodenalis* cysts and *Cryptosporidium* spp. oocysts in green leafy vegetables marketed in the city of Valencia (Spain). Int. J. Food Microbiol..

[bb0885] Troeger C., Blacker B.F., Khalil I.A., Rao P.C., Cao S., Zimsen S.R. (2018). Estimates of the global, regional, and national morbidity, mortality, and aetiologies of diarrhoea in 195 countries: a systematic analysis for the global burden of disease study 2016. Lancet Infect. Dis..

[bb0890] Turasan H., Kokini J. (2021). Novel nondestructive biosensors for the food industry. Annu. Rev. Food Sci. Technol..

[bb0895] Ullah N., Bruce-Tagoe T.A., Asamoah G.A., Danquah M.K. (2024). Multimodal biosensing of foodborne pathogens. Int. J. Mol. Sci..

[bb0900] Useche E., Jiménez A., Armada K., Castillo B., Viettri M., Bandes A. (2022). Standardization of the PCR technique for the detection of the *Toxoplasma gondii* B1 gene in meat and water samples and cloning of the product for use as control. Acta Parasitol..

[bb0905] Vaidya A.M., Annapure U.S. (2019). Enzymes in Food Biotechnology.

[bb0910] Vinayak S. (2020). Recent advances in genetic manipulation of *Cryptosporidium*. Curr. Opin. Microbiol..

[bb0915] Vinayak S., Jumani R.S., Miller P. (2020). Bicyclic azetidines kill the diarrheal pathogen *Cryptosporidium* in mice by inhibiting parasite phenylalanyl-tRNA synthetase. Sci. Transl. Med..

[bb0920] Wang B., Wang H., Lu X., Zheng X., Yang Z. (2023). Recent advances in electrochemical biosensors for the detection of foodborne pathogens: current perspective and challenges. Foods.

[bb0925] Wang L., Zhang Z., Liu Y., Lin S., Zhang W., Zhu L. (2024). Allosteric strand displacement isothermal amplification for the visual detection of *Toxoplasma gondii* in 30 minutes. Biosens. Bioelectron..

[bb0930] Wato T. (2020). The agricultural water pollution and its minimization strategies—a review. J. Resour. Dev. Manag..

[bb0935] Weigum S.E., Castellanos-Gonzalez A., White A.C., Richards-Kortum R. (2013). Amplification-free detection of *Cryptosporidium parvum* nucleic acids with the use of DNA/RNA-directed gold nanoparticle assemblies. J. Parasitol..

[bb0940] WHO (2021). https://www.who.int/data/gho.

[bb0945] WHO, U.N (2015). https://www.who.int/publications/i/item/9789241565165.

[bb0950] Wu W., Wu Z., Yu T., Jiang C., Kim W.-S. (2015). Recent progress on magnetic iron oxide nanoparticles: synthesis, surface functional strategies and biomedical applications. Sci. Technol. Adv. Mater..

[bb0955] Wu Y., Wang C.-W., Wang D., Wei N. (2021). A whole-cell biosensor for point-of-care detection of waterborne bacterial pathogens. ACS Synth. Biol..

[bb0960] Xia F., Zuo X., Yang R., Xiao Y., Kang D., Vallée-Bélisle A. (2010). Colorimetric detection of DNA, small molecules, proteins, and ions using unmodified gold nanoparticles and conjugated polyelectrolytes. Proc. Natl. Acad. Sci..

[bb0965] Xiao X., Li H., Zhao L., Zhang Y., Liu Z. (2021). Oligonucleotide aptamers: recent advances in their screening, molecular conformation and therapeutic applications. Biomed. Pharmacother..

[bb0970] Xu S., Zhang C., He L., Wang T., Ni L., Sun M. (2013). DNA detection of *Toxoplasma gondii* with a magnetic molecular beacon probe via CdTe@Ni quantum dots as energy donor. J. Nanomater..

[bb0975] Yasmin J., Ahmed M.R., Cho B.-K. (2016). Biosensors and their applications in food safety: a review. J. Biosyst. Eng..

[bb0980] Yu F., Zhang K., Wang Y., Li D., Cui Z., Huang J. (2021). CRISPR/Cas12a-based on-site diagnostics of *Cryptosporidium parvum* IId-subtype-family from human and cattle fecal samples. Parasit. Vectors.

[bb0985] Zeng Y., Zhou J., Sang W., Kong W., Qu J., Ho H.-P. (2021). High-sensitivity surface plasmon resonance imaging biosensor based on dual-wavelength differential method. Front. Chem..

[bb0990] Zhang J., Wang Y., Lu X. (2021). Molecular imprinting technology for sensing foodborne pathogenic bacteria. Anal. Bioanal. Chem..

[bb0995] Zhang S., Lin S., Zhu L., Du Z., Li J., Wang L. (2022). Novel indicator and stem-loop-primer assisted isothermal amplification for the visual semi-quantitative detection of *Toxoplasma gondii*. Sens. Actuators B.

[bb1000] Zhang J., Ma C., Du Y., Huang J., Xue L. (2025). Microfluidic biosensors for rapid detection of foodborne pathogenic bacteria: recent advances and future perspectives. Front. Chem..

